# Adverse Birth Outcomes Related to NO_2_ and PM Exposure: European Systematic Review and Meta-Analysis

**DOI:** 10.3390/ijerph17218116

**Published:** 2020-11-03

**Authors:** Valentin Simoncic, Christophe Enaux, Séverine Deguen, Wahida Kihal-Talantikite

**Affiliations:** 1 LIVE UMR 7362 CNRS (Laboratoire Image Ville Environnement), University of Strasbourg, 67000 Strasbourg, France; christophe.enaux@live-cnrs.unistra.fr (C.E.); wahida.kihal@live-cnrs.unistra.fr (W.K.-T.); 2 EHESP School of Public Health, 35043 Rennes, France; Severine.Deguen@ehesp.fr; 3 Department of Social Epidemiology, Institut Pierre Louis d’Epidémiologie et de Santé Publique (UMRS 1136), Sorbonne Universités, UPMC Univ Paris 06, INSERM, 75646 Paris, France

**Keywords:** systematic review, meta-analysis, birth weight, low birth weight, preterm birth, exposure, air pollution, PM, NO_2_

## Abstract

There is a growing number of international studies on the association between ambient air pollution and adverse pregnancy outcomes, and this systematic review and meta-analysis has been conducted focusing on European countries, to assess the crucial public health issue of this suspected association on this geographical area. A systematic literature search (based on Preferred Reporting Items for Systematic reviews and Meta-Analyses, PRISMA, guidelines) has been performed on all European epidemiological studies published up until 1 April 2020, on the association between maternal exposure during pregnancy to nitrogen dioxide (NO_2_) or particular matter (PM) and the risk of adverse birth outcomes, including: low birth weight (LBW) and preterm birth (PTB). Fourteen articles were included in the systematic review and nine of them were included in the meta-analysis. Our meta-analysis was conducted for 2 combinations of NO_2_ exposure related to birth weight and PTB. Our systematic review revealed that risk of LBW increases with the increase of air pollution exposure (including PM_10_, PM_2.5_ and NO_2_) during the whole pregnancy. Our meta-analysis found that birth weight decreases with NO_2_ increase (pooled beta = −13.63, 95% confidence interval (CI) (−28.03, 0.77)) and the risk of PTB increase for 10 µg/m3 increase in NO_2_ (pooled odds ratio (OR) = 1.07, 95% CI (0.90, 1.28)). However, the results were not statistically significant. Our finding support the main international results, suggesting that increased air pollution exposure during pregnancy might contribute to adverse birth outcomes, especially LBW. This body of evidence has limitations that impede the formulation of firm conclusions. Further studies, well-focused on European countries, are called to resolve the limitations which could affect the strength of association such as: the exposure assessment, the critical windows of exposure during pregnancy, and the definition of adverse birth outcomes. This analysis of limitations of the current body of research could be used as a baseline for further studies and may serve as basis for reflection for research agenda improvements.

## 1. Introduction

Low birth weight (LBW) is defined by the World Health Organization (WHO) as birth weight less than 2500 g (referenced P07.0–P07.1 in the 10th revision of the international classification of diseases–ICD 10) [1]. In addition, preterm birth (PTB) is defined as childbirth occurring at less than 37 completed weeks or 259 days of gestation (referenced P07.2–P07.3 in ICD 10). The WHO estimated that between 15% and 20% of births worldwide are LBW, representing 20 million births a year [1]. On the other hand, it is estimated that more than 15 million babies are born preterm every year, more than 1 in 10 babies around the world [2]. In developed countries, PTB rates have been reported to range from 5% to 7% of live births [3]. Moreover, these figures appear to be on the rise [4]. For European countries, according to the European Perinatal Health Report, low birthweight babies accounted for less than 4.5% of all births in Iceland, Sweden, Finland, etc. and around 10% in Spain, France, etc. [5]. The percentage of low birthweight babies was significantly higher in 2015 compared with 2010 in some countries. Comparisons in the preterm birth rate in 2010 and 2015 differed widely between countries significantly higher in 8 countries.

The consequences of LBW and PTB include fetal and neonatal mortality, and morbidity (60% to 80% of all neonatal deaths [6]), poor cognitive development and an increased risk of chronic diseases later in life [7,8,9]. Recent studies have demonstrated that LBW will increase the risk of diabetes and cardiovascular disease later in life (reduction in risk per kg increase in birthweight in both men: hazard ratio (HR) = 0.88, 95% confidence interval (CI): 0.84–0.91, and women: HR = 0.88, 95% CI: 0.82–0.95 [10]). PTB has long-term adverse consequences for health, too. It is well documented that children who are born prematurely are more likely to present cerebral palsy, sensory deficits, learning disabilities and respiratory illnesses compared to children born at term [2,11,12,13,14,15,16,17,18]. Complications related to PTB are the leading cause of death for children under 5 years old, causing an estimated 1 million deaths worldwide in 2015 [6,19]. Therefore, with a range of both, short- and long-term consequences, LBW and PTB represent still today a major public health issue. Additionally, adverse consequences related to LBW and PTB contribute largely to the global health costs [1,19,20,21,22]. According to the European Union (EU) benchmarking report 2009/2010, the statistical data collected from 14 European countries demonstrate the significant and growing cost of prematurity in Europe. For instance, in Denmark each preterm birth cost near 55,460 euros for premature treatment and in France prematurity cost more than 1.5 Billion euro each year. [23].

Risk factors of PTB and LBW are still not completely understood, although the etiology is thought to be multifactorial [2]. It remains unclear whether these adverse outcomes could result from determinants which act independently or in combination. These factors include medical conditions of the mother or fetus, genetic influences, infertility treatments, behavioral and socioeconomic factors, iatrogenic prematurity, and environmental exposure [4,24,25]. Epidemiological studies indicate that currently the ambient air pollution could constitute an important environmental public health issue for individual and public health point of view [26]. In the last decade, a growing body of evidence has associated exposure to ambient air pollution, mainly particulate matter (PM) and nitrogen dioxide (NO_2_), during pregnancy with adverse pregnancy outcomes, especially fetal growth and gestational duration [27,28,29,30,31,32,33]. Pregnancy may constitute a particular period of high susceptibility to pollutants contained in air pollution because of a high level of cell proliferation, organ development and the changing capabilities of fetal metabolism [34]. Molecular studies have provided reasonable biological mechanisms for the association between air pollution and fetal growth and development [35,36]. Ambient air pollution exposure is hypothesized to affect the fetus either directly through trans-placental exposure or indirectly by affecting physiological changes in the mother [37]. Although effects of ambient air pollution on general population, and on pregnancy specifically are relatively small, larger population attributable health risks may be expected due to the ubiquitous nature of ambient air pollution exposure and because all the population is in contact with ambient air and so all the population can be considered at risk [26]. Therefore, it is important that appropriate policies are adopted to diminish ambient air pollution emissions and to raise the awareness of pregnant women [38]. According to the WHO, the goal is to achieve a 30% reduction of the number of infants born with a weight lower than 2500 g by the year 2025 [39]. This would translate into a 3.9% relative reduction per year between 2012 and 2025 and a reduction from approximately 20 million to about 14 million infants with low weight at birth [1].

Based on this evidence, several environmental public health measures have been adopted and implemented at the individual and population level to improve the quality of ambient air, such as promoting cleaner fuel sources and energy technologies, promoting smarter urban planning that aims to reduce urban density and traffic-related pollution, etc. [40]. So far, environmental policies designed to reduce air pollution issue have shown to be effective, with health benefits and helping to reach health policy objectives [41,42]. For instance: Japanese legislation has limited transportation-related emission since 2001. The average NO_2_ concentration decreased from 30 to 21 ppb and PM_2.5_ concentrations decreased from 38 to 26 mg/m^3^. These reductions respectively led to 1.1% and 0.6% lower prevalence of pediatric asthma [43].

To date, Health Impact Assessments (HIA) are recognized to play a crucial role in evaluating different policy scenarios for reducing air-pollution levels; in assessing new air-quality directives; or in calculating the external monetary costs of air pollution or the benefits of preventive actions [44,45]. More precisely, an HIA in this field provides the number of health events attributable to air pollution in the target population [45] and, thereby, in our case, quantifies the air pollution burden of disease due to adverse birth outcomes as preterm birth and low birth weight complications in Europe [46]. Assessment of environmental burden of disease enable the identification of policy priorities. To implement a HIA, several data sources are needed, including the dose-response function; this function derives from epidemiological studies assessing statistical indicator as relative risk associated with the modelled and observed exposure [47]. In our case, this relative risk may come from Europe based meta-analysis providing pooled estimates. One substantial input of meta-analysis is to offer estimates within a specific vulnerable population as well as a closer match with the geographical context of exposure [48]. More often, the dose–response curve linking air pollution and health impacts is supposed to be linear which means that reductions in air-pollution levels, will have consequences for health effects independently to the starting point on the curve. Therefore, this linear relationship cannot capture the different level of an individual’s susceptibility to air pollution [49,50]. It is a reason why preventive action aimed at reducing air-pollution levels in general and not only focusing on air-pollution peaks. Focusing on the peaks of air pollution would only prevent a small number of health events [45].

Recently, there has been a growing number of studies investigating the relationship between adverse birth outcomes, as PTB and LBW, and air pollutant concentration. The possible effect of air pollution exposures on birth outcomes has been reviewed in several systematic reviews and meta-analyses [26,37,48,51,52,53,54,55,56,57,58]. To the best of our knowledge, no European systematic review was performed to consider more homogeneous level of exposure to air pollution. The European Union and WHO have drafted a legislative framework which establishes health-based standards and objectives for several air pollutants. For instance, the threshold for the particulate matter (PM_10_) concentrations is 40 µg/m^3^ on 1 year, for PM_2.5_ 25 µg/m^3^on 1 year, for NO_2_ 40 µg/m^3^ on 1 year and for SO2 it is 125 µg/m^3^ on 24 h, these regulations differ from one continent to another. In this way, the average concentration of various air pollutants differs from one country to another. For instance, the level of exposure to annual average concentration of NO_2_ in the countries of the world, between 2000 and 2015 varied from 97 µg/m^3^ (NYC, USA) and 55 µg/m^3^ (Beijing, China) into 35 µg/m^3^ (Paris, France) and 26.1 µg/m^3^ (Valencia, Spain) [59,60,61,62].

In this setting, updating the literature synthesis of European studies may improve our understanding of the relationship between air pollution, and PTB (as well as LBW). Therefore, we conducted a meta-analysis to assess the association between air pollution and the risk of PTB and LBW, separately, in order to suggest future directions for European research and public health policies.

Our work investigated the following epidemiological question: among newborn in European countries, is air pollution exposure of women during pregnancy significantly related to a risk of adverse birth outcome including weight and term of birth in observational studies?

We focused our analysis only the European studies which investigated the relationship between PM and NO2 and birth outcome—LBW and PTB—in order to produce an appropriate dose-response function within a specific European population as well as a closer match with the geographical context of exposure. Therefore, our European meta-analysis could go beyond the main limitation of HIAs performed today to quantify the environmental burden of disease.

## 2. Materials and Methods

### 2.1. Search Strategy

The systematic literature search was conducted with the PubMed platform in order to access to the Academic Search Complete databases and Medline, among articles published up until 1 April 2020. The search strategy followed the Preferred Reporting Items for Systematic reviews and Meta-Analyses (PRISMA) guidelines [63] and was performed with the following keywords found in article titles and/or abstract:

“ambient air pollution” OR “outdoor air pollution” OR “atmospheric air pollution” AND “birth outcomes” OR “pregnancy outcomes” OR “low birth weight” OR “birth weight” OR “low-birth-weight” OR “birthweight” OR “birth-weight” OR “preterm birth” OR “gestational age” OR “LBW” OR “PTB” AND “Europe” OR “European” OR “Austria” OR “Belgium” OR “Bulgaria” OR “Croatia” OR “Cyprus” OR “Czech Republic” OR “Denmark” OR “Estonia” OR “Finland” OR “France” OR “Germany” OR “Greece” OR “Hungary” OR “Ireland” OR “Italy” OR “Latvia” OR “Lithuania” OR “Luxembourg” OR “Malta” OR “Netherlands” OR “Poland” OR “Portugal” OR “Romania” OR “Slovakia” OR “Slovenia” OR “Spain” OR “Sweden” OR “United Kingdom”

### 2.2. Studies Selction Strategy

**Figure 1:**
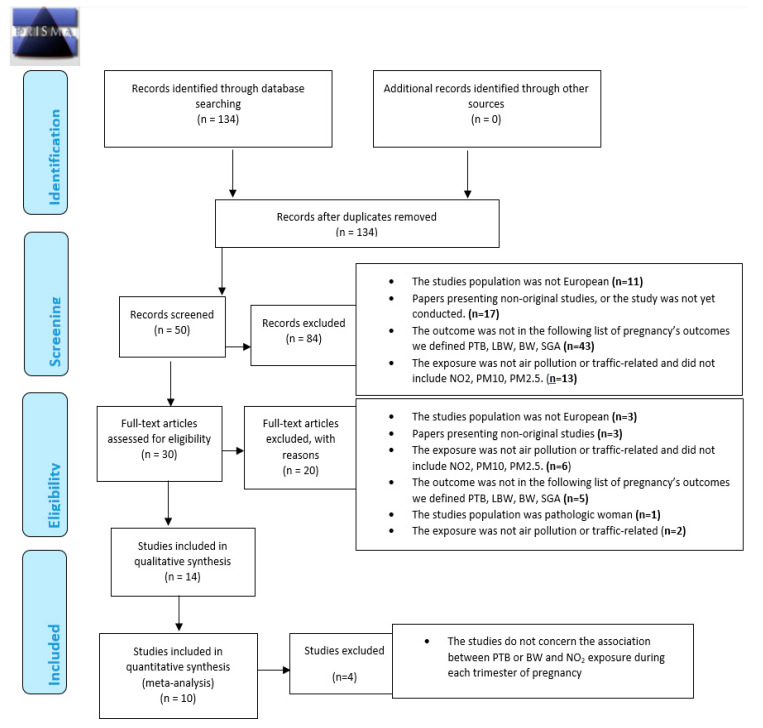
Preferred Reporting Items for Systematic reviews and Meta-Analyses (PRISMA) 2009 Flow Diagram. From: Moher D, Liberati A, Tetzlaff J, Altman DG, The PRISMA Group (2009). Preferred Reporting Items for Systematic Reviews and Meta-Analyses: The PRISMA Statement. PLoS Med 6: e1000097. doi:10.1371/journal.pmed1000097 [63].

Figure 1 summarizes the different steps of the selection process, in line with PRISMA recommendations.

At the first step, the inclusion criteria were human studies, peer-reviewed papers written in English and articles published after 1998.

We restricted our systematic review on geographical location with European study only—for the reason described above—on the pregnant women and pregnancy outcomes, and on ambient air pollution. Papers presenting non-original studies were ultimately excluded.

At the second step, the inclusion criteria were specific pregnancy outcomes definitions including birthweight, low birth weight, preterm birth or small for gestational age (SGA). Secondary criteria were studies investigated specific outdoor air pollutants measured including NO_2,_ PM_10_, PM_2.5_.

Two authors (VS and WK) independently screened the papers based on information in the title, abstracts and full manuscripts to select those papers considered relevant based on the screening criteria described below

At the last step, to perform meta-analysis, among articles included according to the inclusion criteria for the systematic literature review, the inclusion criteria were studies with measure of association between pollutant concentration and birth outcome.

In the last step, bibliographic reference lists of all included studies were searched manually to identify additional studies cited by the previous references.

Finally, meta-analysis was not performed when less than four studies were available for measures of association between a given outcome and a pollutant. Consequently, of the 14 articles included in this systematic literature review, 4 were excluded according to the inclusion criteria for the meta-analysis. Finally, 10 articles were included in the meta-analysis.

### 2.3. Data Extraction

For each study, we extracted and reported in several tables the following information:

- General information: first author’s name, country of origin and date of study;
- Main study characteristics: study design, period, location, statistical methods, population size, main findings (related to PTB, LBW, BW, SGA outcomes and NO_2,_ PM_10_ and PM_2.5_ only);
- Participants’ characteristics: information on confounders, exposure measure;
- Outcome measures (definition, outcomes classification and source).

Assessments of association including odds ratios (ORs), hazard ratios (HRs), relative risks (RRs) and other metrics measuring the strength of association between outcomes and exposure to different pollutants including NO_2,_ PM_10_, PM_2.5_ were extracted. When several measures of association were available, we reported those one from the fully adjusted models.

The two authors (VS and WK) independently extracted all data from selected studies.

### 2.4. Meta-Analysis

When at least four studies were available, the pooled estimate between pregnancy outcomes and exposure to air pollutant was computed. Studies’ risk and beta estimates were expressed as unit corresponding to an increase of 10 µg/m^3^. A fixed or random model based on the Cochran Q-test, the I-square statistic, and the associated *p*-value, was used to obtain the combined effect. The level of heterogeneity between studies is quantified with the I-square indicator (I^2^). When the Cochran Q-test do not reveal significant heterogeneity between studies, a fixed model was applied; inversely, a random model was implemented when the Cochran Q-test was significant. Q-test value between 25% and 50% correspond to a low level of heterogeneity, between 50% and 75% a medium level of heterogeneity and >75% corresponds to a high level of heterogeneity. Forest plots were used to visualize the combined risk estimates. Statistical analysis was performed using the STATA 11 software.

## 3. Results

### 3.1. Studies Selected for Review

In accordance with criteria summarized in Figure 1, in all 134 published selected, a total of 84 studies were excluded based on titles. At the second step, titles of the 134 were screened by two authors (VS and WK) independently. A total of 84 studies were excluded based on the criteria described above. At the third step, the abstracts of the remaining 50 articles (of the 134 articles initially selected) were thoroughly read independently by two experts (VS and WK, authors of this article); 16 were then excluded following criteria described above.

Full manuscripts of the remaining 30 articles (of the 134 articles initially selected) were thoroughly read and 16 articles were excluded. Finally, a total of 14 articles were included according to the inclusion criteria for the systematic literature review. Finally, bibliographic reference lists of all included studies were searched manually to identify additional studies cited by the previous references. No additional article was found. Selected studies are defined in Table 1.

In order to perform a meta-analysis, studies were excluded where there was with a measure of exposure not expressed as a pollutant concentration (for instance: exposed/not exposed) or without measure of association, or when the outcome or the exposure (NOx in summer season) was not pertinent for the meta-analysis.

At last, meta-analysis was performed when at least four studies were available for measures of association between a given outcome and a pollutant. Consequently, of the 14 articles included in this systematic literature review, 4 were excluded according to the inclusion criteria for the meta-analysis. Finally, 10 articles were included in the meta-analysis.

### 3.2. General Description

There were 30 studies published since 1998, including more than 47,805 low birth weight newborns (and subtypes), 311,432 preterm birth (and subtypes) and 3319 newborns small for gestational age, in order to estimate the association between adverse pregnancy outcomes and exposure to three ambient pollutants, NO_2,_ PM_10_ and PM_2.5_. About 10 were eligible for the meta-analyses with the exclusion of 4 studies [75,81,89,91]. Of these, LBW, VLBW, ELBW, PTB, VPTB, EPTB, SGA, gestational age and birth weight were investigated (Table 1). About 4046 cases of preterm birth were included in the meta-analyses and 12,502 births were used to study the birth weight.

### 3.3. Study Design and Location

Most of the studies (10 studies) were conducted in Spain [67,68,69,71,75,77,81,84,85,92]. There were also 5 studies conducted in France [72,76,78,90,91], 2 studies in: Scotland [79,88], Italy [74,89], England [65,80] and only one study in Germany [66], Norway [70], Lithuania [64], Finland [93], Belgium [82]. In addition, two studies included several (more than 10) European countries [73,86], one study included Spain and Belgium [83] and one study included Spain and Italy [87]. Our systematic review group different study designs: the majority of the studies are cohort studies [66,67,68,69,71,72,73,75,76,78,80,82,83,85,86,88,93]; others are ecological time-series studies [64,65,70,74,77,81,84,87,92], spatial approach study [90] and longitudinal study [79]. Prospective study [89], retrospective study [91].

### 3.4. Cases Definition and Data Sources

Several studies investigated the birth weight [66,67,68,70,72,76,80,82,88,89] or gestational age [67], but most investigated specific pathological outcomes. First, several studies investigated LBW and subtypes [64,66,70,73,75,81,84]. Several studies investigated PTB and subtypes [64,65,69,74,77,78,79,81,85,87,90,92,93]. Finally, some studies investigated SGA: birth weight or length below the 10th percentile according to standard percentile charts for sex and gestational age in the population [68,70,75,82,91] (Table 2).

Databases were drawn mainly from birth certificate information and health database from hospital information systems while other form institutes of national health statistics and cohort databases were also used.

### 3.5. Pollutants Investigated

Most frequently, the studies investigate exposure to air pollutants separately NO_2,_ PM_10_ and PM_2.5_ [70,73,75,77,80,86,88] or exposure to PM_2.5_ and NO_2_ [66,81,84,93] or exposure to PM_10_ and NO_2_ [72,74,76,79,82,87,92] or exposure to NO_2_ [64,67,68,69,71,78,83,85,90,91] or exposure to PM_10_ [65,89]. Some papers have used a monitoring station-based approach with average from all monitoring stations [89,92] or average from existing monitoring stations [64,65,74,77,81,84,87] but most used a modeling-based approach with, on the one hand, land-use regression, LUR [66,67,68,69,71,73,75,76,78,80,83,85,86], and on the other hand dispersion models [70,72,76,79,88,90,91]. Few studies use other models as spatial temporal interpolation method (Kriging method) [82] and system for integrated modeling of atmospheric composition (SILAM model) [93].

Table 3 describes the approaches used to assess the residential exposure measures and level of exposure assigned to the population of all studies included in the systematic review (n = 30).

### 3.6. Window of Exposure

The different definitions of critical windows of exposure considered in the 30 studies included in the systematic review is described in Table 4. Short- and long-term exposure to air pollutants were used to investigate the relationship between LBW, PTB, SGA and residential exposure (at home address). Long-term exposures were the most explored cumulative exposure windows [64,66,67,68,69,70,71,72,73,75,76,80,81,82,83,84,85,86,87,88,89,91,92,93]. Moreover, few studies chose not to focus on a particular window of exposure, instead measuring annual average pollutant concentrations at residence [78,79,90].

### 3.7. Overview of Current Evidence Concerning Possible Effects on Birth Outcomes of Exposure to Air Pollution

**Figure 2:**
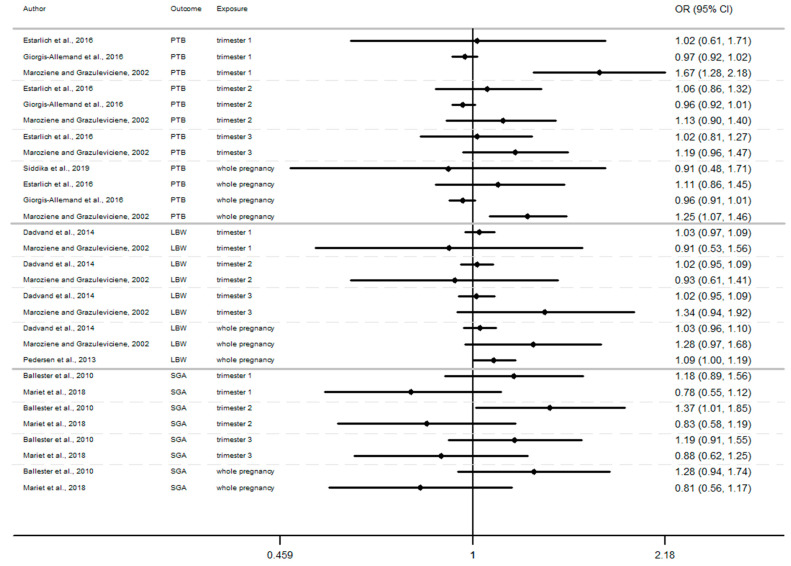
Risk of birth outcome for NO_2_ exposure during different windows of exposure during pregnancy.

**Figure 3:**
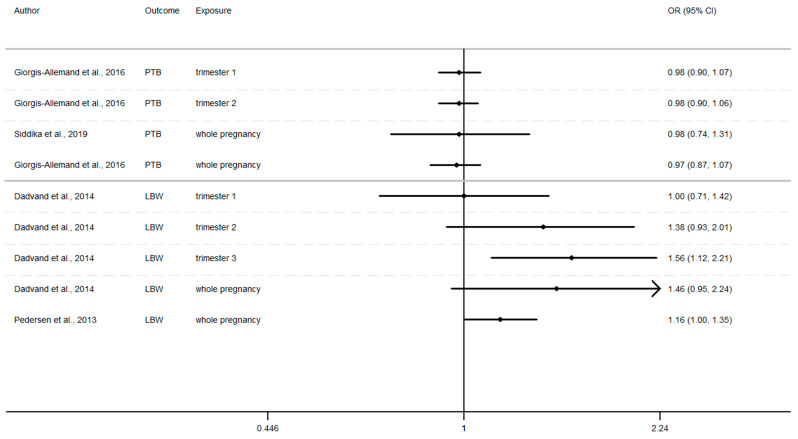
Risk of birth outcome for PM_10_ exposure during different windows of exposure during pregnancy.

**Figure 4:**
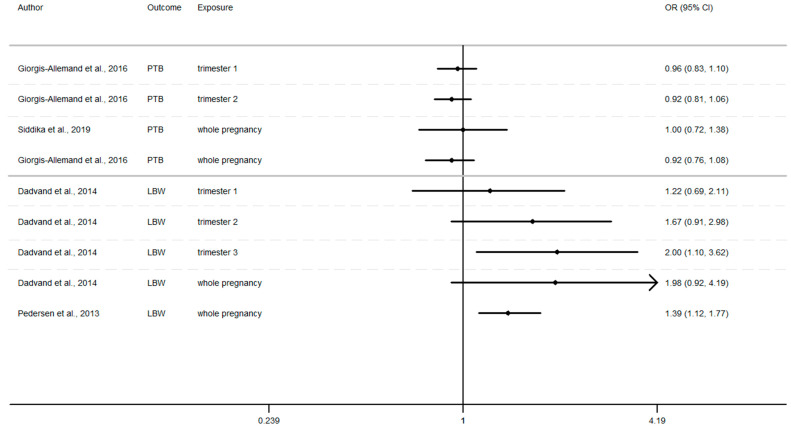
Risk of birth outcome for PM_2.5_ exposure during different windows of exposure during pregnancy.

In this section, the results of studies are presented in Figure 2, Figure 3 and Figure 4, structured by window of exposure of different pollutants (NO_2_, PM_10_, PM_2.5_). Overall, results show the risk of adverse birth outcomes increases for a 10 µg/m^3^ increase NO_2_ exposure. Therefore, 19 results tend to show an association between the increase of risk of adverse pregnancy outcomes and NO_2_ exposure while 10 results which tend to show a decrease of these risks. Our review reveals that for 10 µg/m^3^ increase in NO_2_ exposure (Figure 2) newborn have increased risk of:

- Preterm birth (OR = CI 95%) OR = 1.67 (1.28–2.18) [64] for the first trimester, OR = 1.06 (0.86–1.32), 1.13 (0.90–1.40) [64,85] for the second trimester, OR = 1.19 (0.96–1.47) [64] for the third trimester.
- Small for gestational age (OR = CI 95%) OR = 1.18 (0.89–1.56), OR = 1.37 (1.01–1.85), OR = 1.19 (0.91–1.56), respectively for the windows of exposure of 1st, 2nd and 3rd trimester [68],
- Low birth weight (OR = CI 95%) OR = 1.03 (0.97–1.09), OR = 1.02 (0.95–1.09), OR = 1.34 (0.94–1.92) respectively for the windows of exposure of 1st, 2nd and 3rd trimester [64,73,75].

Furthermore, as shown in Figure 2, Figure 3 and Figure 4 (Appendix A), the LBW risk increases for outdoor air pollutant exposure during the windows of exposure of whole pregnancy.

- a 10 µg/m^3^ increase NO_2_ exposure: OR = 1.03 (0.96–1.10), OR = 1.28 (0.97–1.68), OR = 1.09 (1.00–1.19) [64,73,75].
- a 10 µg/m^3^ increase PM_10_ exposure OR: = 1.46 (0.95–2.24) and 1.16 (1.00–1.35) [73,75]
- a 10 µg/m^3^ increase PM_2.5_ exposures OR: = 1.98 (0.92–4.19) and 1.39 (1.12–1.77) [73,75]

However, several results were not significant, except studies [64,68,75].

Among studies focusing on critical windows, during each window of exposure the number of results which tend to show an association between PTB or SGA and air pollutant are the same and do not increase or decrease with the trimester of pregnancy, for any windows of exposure only three results tend to show an association [64,68,85].

Whereas the risk of LBW seems to increase as the pregnancy progresses. In this way, our review reveals that two results tend to show an association between the risk of LBW and air pollutant exposure (NO_2_, PM_10_, PM_2.5_, Figure 2, Figure 3 and Figure 4) during the first trimester of pregnancy (Appendix B), three results tend to show this association during the second trimester of pregnancy (Appendix C) and four results tend to show this association during the third trimester (Appendix D).

In addition, when studies consider the exposure of the entire pregnancy, seven results found an association between air pollutant exposure and the increase of the risk of LBW against only 3 results for PTB and SGA in the same windows (Appendix A).

Among studies focusing on the 1st trimester of exposure the risk of adverse birth outcomes ranges from 0.78 to 1.67 with confidence interval range from 0.53 to 2.18. For the 2nd trimester of exposure results (OR) range from 0.83 to 1.67 with a confidence interval range from 0.58 to 2.98. For the 3rd trimester of exposure results (OR) range from 0.88 to 2.00 with a confidence interval range from 0.62 to 3.62. These inconsistent results illustrate the lack of uniformity in the methods employed, difference between cross section, variability of variable’s definition, and the lack of studies, particularly in Europe.

For studies focusing on the whole pregnancy for the relationship between pregnancy adverse outcomes risk and air pollutant exposition: NO_2_ [64,68,73,75,85,86,91,93], PM_10_ [73,75,86,93], PM_2.5_ [73,75,86,93], only two studies had significant results. Maroziene and Grazulviciene, 2002 [64] suggest that the risk of PTB increases with NO_2_ exposure (OR = 1.25; 1.07–1.46), while Pedersen et al., 2013 [73] found increased LBW risk with PM_2.5_ exposures (OR = 1.39; 1.12–1.77).

The Pedersen’s study also had nearly significant results for NO_2_ exposure associated with LBW (OR = 1.09; 1.00–1.19) and for PM_10_ exposure associated with LBW (OR = 1.16; 1.00–1.35). Overall, the results reveal that the risk of adverse outcomes including: PTB [64,85,86,93], LBW [64,73,75], SGA [68,91] was not found to be significantly associated with any of the pollutants. As for the other windows of exposure (each pregnancy trimester), results are very heterogeneous and there appears to be no clear trend regardless of the model used. For NO_2_ exposure results (OR) range from 0.81 to 1.28 with a confidence interval range from 0.91 to 1.74. For PM_10_ exposure results (OR) range from 0.97 to 1.46 with a confidence interval range from 0.74 to 2.24. And for PM_2.5_ exposures, results (OR) range from 0.92 to 1.98 with a confidence interval range from 0.72 to 4.19.

## 4. Meta-Analysis

### 4.1. Main Characteristics

The meta-analysis presented in this study was conducted for 2 combinations between one air pollutant and two birth outcomes during different windows of exposure, when at least four studies were available for the same combination. More precisely, the 2 combinations were NO_2_ exposure and related with birth weight and PTB. Table 5 describes the measures of the association of the studies included in the meta-analysis.

In order to differentiate the health effect related to each trimester and entire pregnancy, stratified analyses have been performed, only when this is possible. For the combination between NO_2_ and preterm birth, it was conducted for the entire pregnancy only. Following these conditions, we produced, finally, 5 meta-analyses. Of these, heterogeneity (Q-test) tests indicated one meta-analyses with high I^2^ (I-square indicator) values (above or close to 50%) for which random effects models were applied (for the other four combinations, fixed models were used). Heterogeneity varied from 25.2% to 72.3%, indicating that measurement methods, sample properties, and characteristics varied both among and within different studies.

### 4.2. Birth Weight

**Figure 5:**
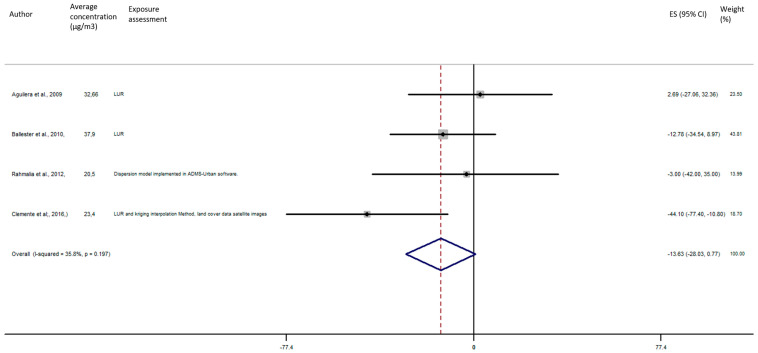
Association between birth weight and NO_2_ exposure during the first trimester of pregnancy.

**Figure 6:**
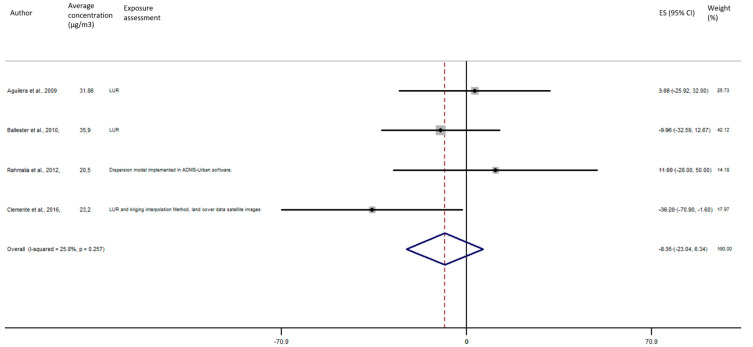
Association between birth weight and NO_2_ exposure during the second trimester of pregnancy.

**Figure 7:**
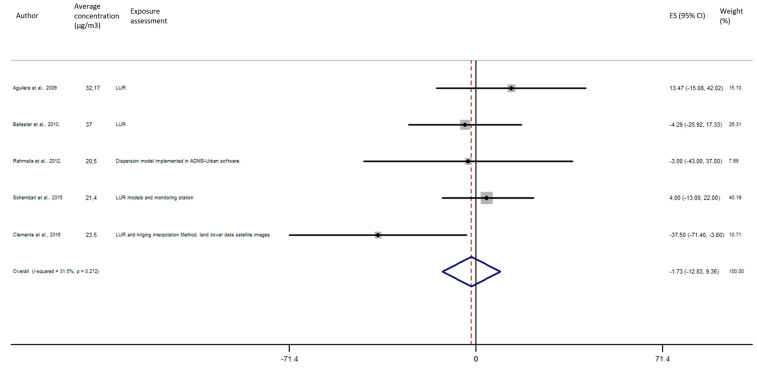
Association between birth weight and NO_2_ exposure during the third trimester of pregnancy.

**Figure 8:**
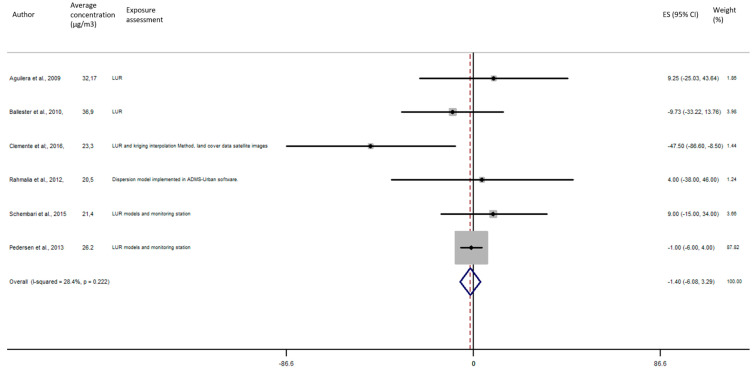
Association between birth weight and NO_2_ exposure during the entire pregnancy.

As shown in Figure 5, the exposure of NO_2_ during any windows of exposure on birth weight was not statistically significant. The overall analysis did not reveal a significant decrease of birth weight in pooled beta for any windows of exposure: for second trimester the pooled beta is: −8.35, 95% CI (−23.04, 6.34) (Figure 6), for the third trimester: pooled beta = −7.04, 95% CI (−19.90, 5.81) (Figure 7). It is interesting to note here that the exposure of NO_2_ during the first trimester tends to show a nearly significant decrease of birth weight in pooled beta = −13.63, 95% CI (−28.03, 0.77). Finally, regarding whole pregnancy, as shown in the Figure 8, the exposure of NO_2_ during the entire pregnancy on birth weight was not statistically significant. The overall analysis did not reveal a significant decrease of birth weight in pooled beta (fixed models: pooled beta = −1.40, 95% CI (−6.08, 3.29)). 

### 4.3. Preterm Birth

**Figure 9:**
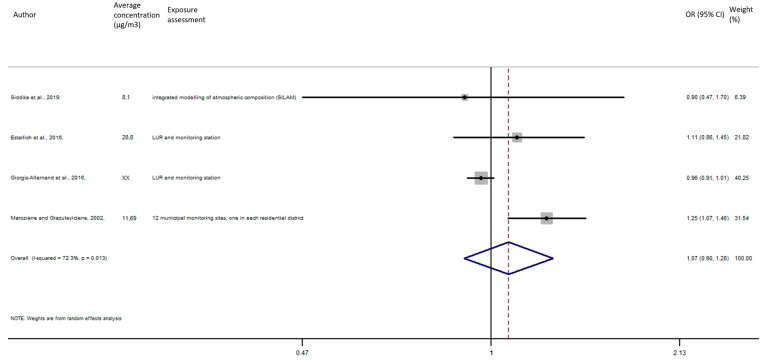
Association between preterm birth and NO_2_ exposure during the entire pregnancy.

As shown in Figure 9, the exposure of NO_2_ during the entire pregnancy on birth weight was not statistically significant, and did not reveal a significant increase of the risk of preterm birth in pooled OR (pooled OR = 1.07, 95% CI (0.90, 1.28)).

### 4.4. Sensitivity Analysis

To estimate the stability of our results, sensitivity analysis was performed by recalculating the pooled effects estimates after omitting one study each time as long as there remained at least 4 studies (Appendix B). We found that the effect estimates of each 10 µg/m^3^ increase in NO_2_ exposure during the entire pregnancy on birth weight showed no significant change by removing one single study, suggesting that the combined results were relatively stable and reliable. This is except for the sensitivity analysis of the association between birth weight and NO_2_ exposure during the third trimester of pregnancy, where the omission of the study of Clemente et al. (2016) [83] induced a reverse of the association that was hitherto negative (Table A1); however, the result was still not statistically significant (beta = 2.5, 95% CI = (−9.18, 14.30)). Small variations were visible, and while point combined estimates were rather similar, the precision level of the confidence interval decreased. 

## 5. Discussion

### 5.1. Main Finding

Our systematic review does not show significant results, but despite this a trend is apparent in that NO_2_ exposure during the whole pregnancy seems to increase the prevalence of LBW. In addition, the result of published European studies included in our systematic review tend to show an increased risk of LBW with a 10 µg/m^3^ increase in PM_2.5_ and PM_10_, specifically for long-term exposure including exposure during last trimester and whole pregnancy. By contrast, no significant excess risk of adverse birth outcomes has been found regardless of pollutant or short-term window of exposure (each trimester).

Our meta-analysis does not reveal a significant result, and the exposure of NO_2_ during the first, second or third trimester on birth weight was not statistically significant. The overall analysis did not reveal a significant decrease of birth weight in pooled beta. For the PTB outcome and the exposure of NO_2_ during the entire pregnancy, the overall analysis did not reveal a significant increase of the risk of preterm birth in pooled-OR.

The characteristics of the different studies (design, adjustment, definition of the outcomes ....) (see Appendix F, Table A3 and Table A4) did not change the meta-risks estimated with the classical meta-analysis approach (data not shown).

These results for long-term exposure converge with international meta-analysis (see results in Appendix G) which show positive correlation between PM_2.5_, PM_10_, NO_2_, exposures during the entire pregnancy and LBW. [94] Conversely, international studies tend to show significant association between LBW and ambient air pollutant also during short-term exposure.

These results could be partially explained by methodological limitations inherent in the heterogeneity of the method of exposure assessment, definition of adverse birth outcome, definition of confounders and critical windows of exposure, thus limiting the number of studies usable in the meta-analysis which can reduce the statistical significance of possible risk.

The main hypotheses for the biological mechanism are that ambient air pollution could cause inflammation, oxidative stress, affect placental growth, decrease placental exchange, lead to endocrine disruption, etc. [95,96]. More specifically, oxidative stress induces DNA damage and mitochondrial DNA damage, and fosters inflammation, which appear to be important mechanisms of fetal growth [83,97,98,99]. Another specific mechanism affects the placenta; the maternal and fetal circulation are separated by the placental barrier; this barrier contains placental transporters that can regulate or facilitate external compounds [100,101]. Transient receptor potential channels are highly expressed in the placenta, and they can be affected by air pollution exposure. Non-human animal studies reveal that these receptors play important roles in placental development and regulating the fetal–maternal interface in mice models [102].

### 5.2. Outcome Data: Case Selection

We identified many pathways whose outcome information can lead to a bias in the assessments of association. Firstly, outcome definition itself could constitute a source of uncertainty and lead to qualification bias. Many studies investigated birth weight [66,67,68,70,72,76,80,82,88,89] or gestational age [67], but most investigated specific pathological outcomes; first, several studies investigated LBW and subtypes (VLBW, ELBW): birth weight <2500 g International Classification of Diseases 10th Revision; ICD-10: P07.0–P07.1 [64,70,73,75,81,84], birth weight <3000 g [65], VLBW between 1500 g and 2500 g [83] and ELBW <1500 g [83]. Several studies investigated PTB and subtypes: birth occurring before the 37th week of pregnancy; ICD-10: P07.2–P07.3 [64,65,69,78,81,85,87,90,92,93] birth occurring between the 33th and the 37th week of pregnancy [79], birth occurring between the 22th and the 36th week of pregnancy [74], birth occurring between the 30th and the 37th week of pregnancy [77], birth occurring before the 33th week of pregnancy [79], birth occurring before the 30th week of pregnancy [77], birth occurring before the 24th week of pregnancy [87]. Last but not least, some studies investigated SGA: birth weight or length below the 10th percentile according to standard percentile charts for sex and gestational age in the population; ICD10 codes in medical records, O36.5, P05.0, P05.1 [68,70,75,82,91]. Databases were drawn mainly from birth certificate information and health database from hospital information systems while other from institutes of national health statistics and cohort database. In addition, the databases used to collect health data including maternal and newborn characteristics are another source of limitation. PTB and LBW were the most frequently investigated outcomes in included studies. This is an expected finding because, according to the WHO, these outcomes are technically simple parameters to monitor prenatal health in a population and have short- and long-term public health implications. Assessment of gestational duration was most often based on the date of last menstrual period, which could introduce misclassification with recall bias depending on postconceptional bleeding, but also, menstrual irregularities, or late access to prenatal care [70].

### 5.3. Confounding Factors

Our findings need to be interpreted with prudence due to weaknesses that could affect the significance of the associations and then the redaction of accurate conclusions. The different adjustment factors used in each study and the different sample size may lead to difficulties between studies comparisons.

Indeed, most studies were adjusted for mothers’ characteristics (smoking during pregnancy, passive smoking during pregnancy, parity, education, race/ethnicity, age, gestational age, height, pre-pregnancy weight, etc.) [64,66,67,68,69,70,71,72,73,74,75,76,78,79,80,82,83,85,86,87,88,89,90,93] Some studies used birth characteristics (sex, birth order, fetal size) [67,68,69,70,71,72,73,75,76,78,80,82,83,85,86,88,89,91,93]. Other used neighborhood characteristic (city, exposure to other air pollutants, socioeconomic status, type and length of roads, population density, land coverage around the home address, temporal variations in pollution during pregnancy…) [66,68,69,70,71,75,76,78,79,82,85,86,87,90,91,93] Some of them used other characteristics like meteorological characteristic (e.g., temperature, humidity, season of conception or birth) [64,65,67,68,69,70,71,72,74,75,76,78,79,80,81,82,83,84,85,86,87,88,89,91,92,93]. Only one study did not use any covariates [77].

### 5.4. Exposure Assessment

Our systematic review revealed that several approaches for exposure assessment during pregnancy were implemented, and this could induce misclassification of exposure. Some papers have used average from monitoring existing stations [64,65,74,77,81,84,87] or a monitoring station-based approach with an average from all monitoring stations [89,92]. The size of the study area and the number of monitoring stations vary between studies and this may increase the level of heterogeneity of air pollution measurement. The number of monitoring stations varied between a minimum of 1 [65,87] and a maximum of 53 [89]. Consequently, there is a risk of bias when a small number of monitoring stations cover a wide area. The weak spatial representativeness of exposure influences the assessment of the residential exposure of pregnant women. Moreover, collection of these data is often based on national air quality guidelines and legislation and thus may not be optimal in the assessment of exposure and use with health data.

Most of the studies used modeling-based approaches with, on the one hand, LUR [66,67,68,69,71,73,75,76,78,80,83,85,86], and on the other hand dispersion models [70,72,76,79,80,81,82,83,84,85,86,87,88,89,90,91]. Few studies use other models as spatial temporal interpolation method (Kriging method) [81] and SILAM model [93]. These models allow to quantify individual levels of exposure and investigate the health consequences of exposure.

Even if modelling is the gold standard for environmental and health impact assessment, some bias may exist. Overall modelling approaches did not consider residential and daily mobility of pregnant women across the study area and thus exposure misclassification may occur. Finally, environmental modelling can hardly be applied for outdoor and indoor pollution concurrently; notably because of a lack of information on the correlation of indoor and outdoor air pollution depends on geographical and meteorological conditions, building types and systems, and air exchange rates [103].

### 5.5. Critical Windows of Exposure

The definition of window of exposure could induce exposure misclassifications. In our systematic review, two main approaches define the window of exposure in order to investigate the relationship between birth outcomes and residential exposure: long-term exposure, and short-term exposure.

To investigate pollutant exposure, studies used diverse windows of exposure, some of them used short-term daily exposure [65,74,77] or short-term cumulative exposure [65,82,87]. For example, different indicators for daily exposure were identified: the day of the birth (Lag0) [65,74], the day before birth (Lag1) [74] or longer lags such as from lag 1 to lag7 [74,77], or from lag 1 to lag 30 [74] (see Table 4). The studies that investigated short-term cumulative exposure examined also different windows of exposure including over 1 days before birth (lag0–1) [65], over 2 days (Lag0–2) [65,87], 3 days (Lag0–3) [65], 4 days (Lag0–4) [65], 5 days (Lag 0–5) [65], over 6 days before death (Lag 0–6) [65] or for the last week of pregnancy (lag 0–7) [82]. Several studies used long-term exposure, based on cumulative exposure during a given period of pregnancy, with diverse windows [82,84,86,91], but most used weekly exposure [81,84,87,88,92], trimester of pregnancy exposure [64,67,68,69,70,71,72,73,75,76,80,82,83,85,89,91,92] and 9 months of pregnancy exposure [64,66,67,68,69,70,71,72,73,75,76,80,83,85,86,87,91,93]. Finally, some studies investigated with no specific windows and used annual exposure [78,79,90] (see Table 4). Previously, certain meta-analysis and systematic reviews have reported that 1st trimester, 3rd trimester and last gestational month may be a possible critical window of exposure for preterm birth [25].

### 5.6. Assessment Approach and Mean Level of Exposure

The results found in the studies selected may vary according to mean level of exposure in each country, and particularly in each area of study. Our systematic review reveals that the risk of adverse birth outcome tends to be higher among study areas with low air pollutant average concentration. However, we highlight that these studies used mainly monitoring station. Some studies tried to estimate the discrepancy between results in the association between air pollution and birth outcome with different methods for estimating exposure [104,105]. They found that the level of NO_2_ during pregnancy estimated by the nearest air quality monitoring station (AQMS) and by the temporally adjusted geostatistical model (TAG), tend to show the same associations [104,105]. For PM, the use of the nearest AQMS or dispersion models indicated consistent results both in terms of exposure estimates and association with birth weight [105]. Studies tend to show that AQMS and kriging rather predict the average level of pollutant in the urban area, whereas local patterns of variation and LUR might be the most robust methods to predict long-term exposure in complex areas [106]. In this way, pertinence of the method used for the exposure assessment mainly depends on the time-window length and endpoints considered, the spatio-temporal variability of the pollutants and the population’s mobility [76].

### 5.7. Limitations and Risk Estimate of Birth Outcome

The features of the studies described above—such as study population, study design, sample size, the classification and definition of infant death, exposure assessment, difference between interquartile (IQR) used to assess the increase of exposure (Appendix H) and confounding factors—could all, independently or in combination, affect the quality of each study itself and, also, their comparison in our systematic review. Some factors may overestimate while other one may underestimate the risk of birth outcome.

The loss of precision inherent to such a general classification scheme (the definition of outcome and included all live birth) may reduce the likelihood of detecting an association between low birth weight and the study exposures. For instance, broad groupings of low birth weight into all LBW including term and preterm birth have also hampered the ability to examine associations for specific LBW by diluting relevant cases.

One source of such limitation lies in the databases. Using linked birth-hospital databases may reduce the likelihood of missing information, because it includes all birth information collected throughout birth, rather than only from institutes of national health statistics and cohort databases. Missing data, if not included, may yield the same effect, so that risk estimates of birth outcome, in particular, may be inaccurate.

In addition, the various confounding factors included in the individual studies make difficult the comparisons between studies. An absence of systematic adjustment for commonly known factors may affect the measure of association and thus the comparisons of all the risk estimates—for instance, folic acid supplementation, or information on dietary factors which are known to decrease the risk of birth outcome. These risk factors tend to vary across the unit of analysis and if they are coincident with the exposure measures, then these spatial confounders will bias the results of the study.

Exposure misclassification may occur where the birth certificate address does not reflect the mother’s true residence during the relevant window of fetal development. To assign exposure, many studies used maternal address at delivery rather than address around conception and during each trimester. This can have a particular impact on studies exploring the risk of birth outcome. Misclassification of exposure may occur following changes in residence during the pregnancy. Some studies revealed that residential mobility among pregnant women between conception and delivery ranged from about 12% in the former to 32% according country. In addition, this residential mobility may vary according to certain individual and contextual characteristics such as age, race, socioeconomic status and other factors including socioeconomic characteristics. This means that the exposure misclassification error due to using delivery address might be greater among younger mothers than among older ones, a phenomenon that might result in confounding—because age is also associated with the risk of poor pregnancy outcome. Therefore, where authors have restricted their analysis to women who resided at the address noted on the medical record before delivery, a slight increase of risk estimate may be observed.

Finally, misclassification of exposure may result from the use of postcode, census block or city level to define the location of maternal residence. These spatial units might not be valid measures of exposure level because they vary considerably in size and are irregular in shape. Therefore, the larger the spatial unit, the more likely it is that bias will be introduced due to heterogeneity within these units, and ecological fallacy may result.

### 5.8. European Versus International Systematic Review: Comparison with Previous International Systematic Reviews

The limited systematic review of European studies may explain the result obtained. Appendix D summarizes the main characteristics and results of previous systematic review and meta-analysis studies selected to compare our results. Most of the earlier reviews were based on cohort design studies like our systematic review. However, all previous systematic review was based on mostly US studies. Similar methodological issues were identified by previous systematic review including outcome and difference in the characterization of exposure and outcome and control of confounding factors.

However, the European average concentration of air pollutants seems to be lower than international average concentrations, moreover the results found in the studies selected may vary according to mean level of exposure in each country, and particularly in each area study. Our results reveal that, in Europe-based studies, the risk of adverse birth outcome tends to be higher among study area with low air pollutant average concentration.

Our findings for long-term exposure converge with international meta-analysis (see results in Appendix D) which show a positive correlation between PM_2.5_, PM_10_, NO_2_, exposures during the entire pregnancy and LBW [94]. Conversely, international studies tend to show significant association between LBW and ambient air pollutant also during short-term exposure.

### 5.9. Limitations

To complement the limitations described earlier, both our systematic review and our meta-analysis, present their own strengths and limitations. First, our search could suffer from study selection biases. Non-English publications of relevant articles may have been ignored. Furthermore, we cannot exclude the possibility that our systematic review could be impacted by publication bias. Indeed, unpublished results (including grey literature and results not statistically significant, which are not available) may influence our meta-analysis findings towards the statistical significance of the risk estimates

## 6. Public Health Implication

To date the main inherent limitation of environmental health risk assessments is related to uncertainties of the assumptions made about the dose–response function. More particularly, the potential limitations of geographic extrapolation of the shape of the risk function may be less well-defined in some geographic areas with the lowest concentrations. In some studies, the authors used the exposure–response function from only one cohort US study [107] while the other one used meta-analysis as a source to estimate the burden [108]. To our knowledge no European meta-analysis permits us to provide a more appropriate source of risk function in order to perform HIAs in European countries with the lowest concentration levels. Thus, the burden could derive from a non-coherent shape risk function that carries larger uncertainties. Our meta-analysis results provide pooled-risk for 5 combinations of air pollutant and birth weight and PTB, which may provide a coherent exposure–response function for environmental health risk assessments in European countries.

## 7. Conclusions

In spite of the limited number of epidemiological studies selected in the present literature review, our finding suggests that an increase air pollution exposure during pregnancy might contribute to adverse birth outcomes, especially LBW. This body of evidence has limitations that impede the formulation of firm conclusions and so new well-focused European studies are called for.

Our findings need to be interpreted with prudence due to weaknesses that could affect the significance of the associations and hence the drawing of accurate conclusions. Further studies, well-focused on European countries, are called for to resolve these limitations; in particular, the definition of the exposure assessment, the critical windows of exposure and the different adverse birth outcomes, which could affect the strength of association. Future studies could be based on this analysis of limitations of the current body of research, which may provide inspiration for research agenda improvements.

## Figures and Tables

**Table 1:** Main characteristics of the selected studies, order by year of publication.

Authors	Study Design, Period Location	Population Size	Outcomes	Pollutants	Statistical Methods	Confounders/Stratification	Main Findings
Maroziene and Grazuleviciene, 2002, [64]	Population based study, Kaunas (Lithuania), 1998	3988 newborns 140 LBW 203 PTB	LBW (<2500 g), PTB (<37 w)	NO_2_,	Multivariate logistic regression	- Maternal characteristic: parity, age, marital status, education, maternal and paternal smoking, - birth characteristics: gestational age - others season of birth	Results suggest significant association between NO_2_ exposure during first trimester and PTB risk.
Lee et al., 2008, [65]	Time-series analysis London (United Kingdom), 1988–2000	482,765 newborns 29,716 PTB	PTB (<37 w)	PM_10_	Regression model	- Other: temperature, rainfall, sunshine, relative humidity, barometric pressure, largest drop in barometric pressure	No significant association had been revealed.
Slama et al., 2007, [66]	cohort, Munich (Germany) Jan 1998–Jan 1999	1016 newborns 142 birth (<3000)	Birth weight (<3000 g)	PM_2.5_ NO_2_	Poisson regression,	- Maternal characteristic: gestational duration, sex, smoking, height, weight, and education	Significant association between increase only in exposure to PM_2.5_ and decrease in term birth weight mainly during the third trimester.
Aguilera et al., 2009 [67]	Cohort study, Sabadel (Spain), between June 2004 and July 2006	570 newborns	Birth weight	NO_2_	Linear regression models	- Maternal characteristic: tobacco smoking during pregnancy, Passive smoking during pregnancy, parity, education, race/ethnicity, age, gestational age, height, pre-pregnancy weight - birth characteristics: child’s sex, - others: season of conception, Paternal height, paternal weight.	Significant association between birth weight exposure to NO_2_.
Ballester et al., 2010, [68]	Prospective birth cohort, valencia (spain), April, june et nov 2004 and feb 2005.	785 newborns 51 PTB	Birth weight, length, head circumference, SGA	NO_2_	Generalized additive models	- Maternal characteristic: lifestyle variables twice during their pregnancy, maternal age, pre-pregnancy weight, height, gestational weight gain, parity, education, smoking during pregnancy, country of origin, season of last menstrual period - birth characteristics: sex. - neighborhood characteristics: socio-demographic characteristics, - others: environmental exposure, paternal height	Significant association between NO_2_ exposure during the first trimester with birth weight. Significant association between NO_2_ exposure during first and second trimester and SGA.
Llop et al., 2010, [69]	Cohort study, Valencia (Spain), February 2004-June 2005	785 newborns 47 PTB	PTB (<37 w)	NO_2_	Multivariate logistic regression model and multivariate segmented logistic regression model	- Maternal characteristic: age, pre-pregnancy weight, parity, educational level, socioeconomic status, country of origin, working status, cohabitation with the baby’s father, smoking, and the consumption of coffee and alcohol during pregnancy - birth characteristics: sex - neighborhood characteristics: place of residence, - others season of last menstruation	Significant association between PTB and NO_2_ exposure during second, third trimester and entire pregnancy only when women were exposed to NO_2_ levels higher than 46.2 µg/m^3^.
Madsen et al., 2010 [70]	Medical Birth Registry based study, Oslo (Norway), 1999–2002	25,229 newborns 303 LBW 2422 SGA	Birth weight, LBW (term, <2500 g), SGA	NO_2_, PM_10_ PM_2.5_	Logistic regression models, and general linear regression models	- Maternal characteristic: gestational length in weeks, education, smoking status, ethnicity, age, parity. - birth characteristics: sex	No significant association had been revealed.
Estarlich et al., 2011, [71]	Multicenter cohort, Spain, Novomber 2003-February 2008	2337 newborns	Birth weight	NO_2_	Linear regression models	- Maternal characteristic: age, height, pre-pregnancy weight, pre-pregnancy body mass index (BMI), weight gain, education, working status, socioeconomic status, country of origin, cohabitation with the father of the baby, smoking, and environmental tobacco exposure], paternal height - birth characteristics: infant sex - neighborhood characteristics: type of zone (urban vs. rural), - others: season of last menstrual period.	Invers but non-significant association between Increase in NO_2_ during the second trimester and reduction of birth weight.
Rahmalia et al., 2012, [72]	Cohort study, Poitiers, Nancy (France), February 2003-January 2006	1154 newborns	Birth weight,	NO_2_, PM_10_	Linear regression models	- Maternal characteristic: height, pre-pregnancy weight, parity, age at end of education, second trimester smoking, active smoking. - birth characteristics: gestational duration, infant sex, - others: season of last menstrual period, center of recruitment	No significant associated had been revealed.
Pedersen et al., 2013 [73]	Multicenter cohort study, 11 European country, February 1994–June 2011	74,178 newborns 1257 LBW	Term LBW (>37 w And <2500 g) Birth weight	PM_2.5_ PM_10_ NO_2_	Logistic regression models linear regression models	- Maternal characteristic: parity, active smoking, and education - birth characteristics: sex	Significant association between increased risk of low birthweight at term and PM_2.5_ exposure.
Schifano et al., 2013 [74]	Time series analysis, Rome (Italia), 2001–2010	132,691 newborns 847 PTB (22–32 w) 6412 PTB (33–36 w)	PTB (>22 <36 w)	PM_10_ NO_2_	Poisson generalized additive model	- Maternal characteristic: Socio-demographic, long-term trend - others: seasonality and for days of holiday. - Stratification: cold season/warm season	A significant association between PTB and PM_10_ exposure at a lag-period of 12–22 days during the warm season.
Dadvand et al., 2014, [75]	Cohort study, Barcelona (Spain), 2001–2005	6438 newborns 190 term LBW. 803 SGA	Term LBW, SGA	PM_2.5_ PM_10_ NO_2_	Logistic regression models	- Maternal characteristic: ethnicity, education level, marital status, age, smoking during pregnancy, alcohol consumption during pregnancy, body mass index at the time of admission, diabetes status), infection, parity, - birth characteristics: sex of baby, - neighborhood characteristics: neighborhood socio-economic status - others season of conception and year of conception	Significant association between increase in term LBW risk and increase third-trimester exposure to PM_2.5_ and PM_10_.
Sellier et al., 2014 [76]	Cohort study Nancy and Poitiers (France), 2002–2005	1026 pregnant women (PM_10_ study area) 776 pregnant women (NO_2_ study area)	Birth weight (g)	NO_2_ PM_10_	Linear regressions adjusted	- Maternal characteristic: gestational age, height, pre-pregnancy weight, age at the end of education, active and passive smoking during the relevant time-window under study - birth characteristics: sex, birth order - neighborhood characteristics: city - others: month of conception	The association with birth weight tended to be negative with exposure during the 1st trimester of pregnancy, positive with the 2nd trimester of pregnancy and null with the 3rd trimester of pregnancy.
Arroyo et al., 2016 [77]	Time-series analysis, Madrid, 2001–2009	298,705 newborns 24,620 PTB 20,442 VPTB 4178 EPTB	PTB (<37 w) VPTB ([30–37 w]) EPB (<30 w)	PM_2.5_, PM_10_, NO_2_,	Over-dispersed Poisson regression models	- No cofounders/stratification	Significant association between short term exposure to PM_2.5_ and PTB.
Bertin et al., 2015, [78]	Prospective birth cohort, Bretagne (France) 2002–2006	2509 newborns 83 PTB	PTB (<37 W)	NO_2_	Logistic regression models	- Maternal characteristic: high blood pressure before/during pregnancy, gestational diabetes, maternal level of education, fish intake, BMI	Significant increased risk of PTB was associated to exposure to NO_2_ concentrations >16.4 µg m-3 only in urban areas.
Dibben et Clemens, 2015 [79]	Longitudinal study Scotland, 1994–2008	23,086 newborns 21,843 newborns (at term) 457 LBW	LBW (<2500), Birthweight for term births PTB (33–37, <33 W)	NO_2_, PM_10_	Multilevel logistic, linear and multinomial regression model	- Maternal characteristic: age, parity, educational level, social class, ethnicity, lone parenthood, tobacco - neighborhood characteristics: area crime rate - others: season of birth	Increase risk of LBW with the increase of NO_2_ and of PM_10_. Non-significant association of PTB with NO_2_, as well as with PM_10_. Significant association between birthweight for term birth and mean annual levels of NO_2_ and PM_10_.
Schembari et al., 2015 [80]	Cohort study Bradford (England), 2007–2010	9067 newborns	Birthweight (g)	PM_2.5_ PM_10_ NO_2_	Multivariate linear regression models	- Maternal characteristic: ethnicity (for adjust and stratified), age, height, pregnancy weight at first gynecological visit, parity, active smoking during pregnancy, education, and housing tenure - birth characteristics: sex, gestational age, 2-h post load plasma glucose test - others: season of conception	No significant association had been revealed.
Arroyo et al., 2016 [81]	Time-series study, Madrid, 1 January 2001 to 31 December 2009	298,705 newborns 39,583 LBW 24,586 PTB	LBW (<2500 g), prematurity (<37 w)	PM_2.5_, NO_2_	Poisson regression models	- Others: pollinic pollution	A significant association between LBW and exposure to NO_2_ during second trimester. A significant association between LBW and exposure to PM_10_ during second trimester.
Bijnens et al., 2016 [82]	Prospective birth cohort, East Flanders, Belgium, 2002–2013	4760 twins, 2380 PTB 292 VPTB	Birth weight, SGA	PM_10_, NO_2_	Multilevel regression analysis and generalized linear model	- Maternal characteristic: parity, gestational - age (linear and quadratic), age, zygosity and chronicity, maternal age - birth characteristics: sex, birth order, - neighborhood characteristics: neighborhood household income - others: season of birth, birth year	Significant association between higher PM_10_ and NO_2_ exposure during the third trimester and lower birth weight and higher risk of small for gestational age.
Clemente et al.; 2016, [83]	Prospective birth cohort, Spain, (2004–2008), Belgium (2010–2013)	376 newborns (Spain) 550 newborns (Belgium)	Birthweight	NO_2_	Land use regression (LUR). and kriging interpolation Method, land cover data satellite images	- Maternal characteristic: age, ethnicity, parity, smoking status, education, pre-pregnancy maternal BMI - birth characteristics: gestational age, sex, - others: season of birth	Significant association between increase NO_2_ exposure and decrease in birth weight.
Diaz et al., 2016 [84]	Time-series analysis, Madrid (Spain), 2001–2009	298,705 newborns 3290 LBW 1492 term LBW 1176 VBW 236 ELBW	LBW (VLBW: 1500 g to 2500 g and ELBW: <1500 g)	PM_2.5_, PM_10_, NO_2_	Over dispersed Poisson regression models	- Others: controlled for trend and seasonality	Significant association between increase risk of LBW and VLBM and exposure to PM_2.5_ during third months. Significant association between increase risk of ELBW and exposure to PM_2.5_ during eight months. No significant association with the two other pollutants.
Estarlich et al., 2016, [85]	Birth cohort study, November 2003–February 2008, Asturias, Gipuzkoa, Sabadell and Valencia (Spain)	2409 pregnant women 115 PTB	PTB (<37 w)	NO_2_	Logistic regression models	- Maternal characteristic: socio-economic status, active smoking during pregnancy, maternal age - birth characteristics: infant’s sex, - neighborhood characteristics: socio-demographic characteristics, environmental exposures, zone of residence - others: parental season of delivery	No statistically significant associations between exposure to NO_2_ and PTB Significant association between NO_2_ exposure during the second trimester and whole pregnancy and PTB only a woman spending more time at home.
Giorgis-Allemand et al., 2017, [86]	Cohort study, 1994–2001 (ESCAPE, European Study of Cohorts for Air Pollution Effects from 11 European countries)	71,493 newborns 3533 PTB	PTB (<37 w)	PM_2.5_ PM_10_ NO_2_	Logistic regression models with a random effect Survival model a discrete-time Cox model	- Maternal characteristic: age, education, mother alone, parity, smoking, height and weight, pregnancy hypertension - birth characteristics: sex, cesarean delivery - neighborhood characteristics: country - others: meteorological factors, season of conception, outdoor temperature, humidity, and atmospheric pressure,	No significant association.
Schifano et al., 2016, [87]	Population- based pregnancy cohorts, Rome (Italia) 2001–2010, Barcelona (Spain), 2007–2012	78,633 newborns (Rome), 27,255 newborns (Barcelona) 4325 PTB: in Rome 1227 PTB: in Barcelona	PTB (<36 weeks), birth (>22 or >24 w)	PM_10_, NO_2_	Cox regression models	- Maternal characteristic: long time trend, age, education level, age, nationality, eclampsia and chronic pathologies, obstetric diseases in the current pregnancy and chronic diseases in both the current pregnancy and in the past two years - neighborhood characteristics: citizenship - others: seasonality, year	Significant association between PM_10_ and increased risk of PTB in Barcelona and with a decreased risk in Rome. Significant association between decreased risk and exposure to NO_2_.
Clemens et al., 2017, [88]	Cohort study, North-East Scotland, 2002–2011	13,775 newborns, 12,467 mothers	Birthweight	PM_2.5_, PM_10_, NO_2_	Mixed effects regression models	- Maternal characteristic: age at delivery, parental social class, parity, height and weight in early pregnancy, smoking - birth characteristics: sex - others: year of scan	Significant association between exposure to PM_10_, only and reduction of birthweight.
Giovannini et al., 2017, [89]	Prospective study, lombardia (Italia), January 2004–December 2006	3614 women	Birth weight (g), placental weight, umbilical artery PH	PM_10_	Linear regression model	- Maternal characteristic: age, educational level, parity, disease in pregnancy (diabetes and hypertension), normal or pathological course of pregnancy, use of medication, pre-pregnancy BMI, weight gain during pregnancy, gestational age - birth characteristics: gender and bimester of delivery - others: number of ultrasounds	Significant negative association between exposure to PM_10_ during the first trimester and Birth weight.
Deguen et al., 2018, [90]	Ecological study, Paris (France), January 2008–December 2011	105,346 newborns 4871 PTB	PTB (≤36 W)	NO_2_	Spatial scan statistic, spatial clustering approach	- Neighborhood characteristics: socioeconomic deprivation index - interaction between socioeconomic deprivation index and NO_2_	Spatial excess risk of PTB was explained by spatial variation of NO_2_ concentrations and socio-economic deprivation.
Mariet et al., 2018, [91]	Retrospective study, Besançon, Dijon (France), 2005–2009	249 multiple pregnancies 506 newborns 94 SGA	fetal growth restriction (FGR), SGA	NO_2_	Multivariable logistic regression and model multilevel model	- Maternal characteristic: age older than 35 years at delivery, smoking during pregnancy, malnutrition, nulliparity, gestational hypertension and diabetes - neighborhood characteristics: low neighborhood socioeconomic level, - others: the adjustment for major infant congenital abnormalities in addition to the 7 previous factors led to the same results	No significant association had been revealed for SGA.
Arroyo et al., 2019 [92]	Time-series analysis, Spain, 2001–2009	1,468,622 newborns 127,722 PTB	PTB (<37 w)	PM_10_, NO_2_	Generalized linear models with link Poisson	- Others: trend, seasonality, temperature in periods of heat and/or cold waves	Significant increase risk of PTB for 10µg/m^3^ increase in NO_2_ and PM_10_.
Siddika et al., 2019, [93]	Population-based cohort study, Espoo (Finland), 1984–1990	2568 newborns 195 PTB	PTB (<37 w)	PM_2.5_, NO_2_	Poisson regression analysis	- Maternal characteristic: age, smoking during pregnancy, exposure to environmental tobacco smoke during pregnancy, single parenthood - birth characteristics: sex - neighborhood characteristics: exposure to other air pollutants, family’s socioeconomic status	No significant association between PTB and exposure to PM_2.5_, PM_10_ or NO_2._

PM: particulate matter; PM_2.5_: particulate matter with an aerodynamic diameter up to 2.5 μm; PM_10_: particulate matter with an aerodynamic diameter up to 10 μm; NO_2_: nitrogen, LBW: low birth weight, VLBW: very low birth weight, PTB: preterm birth, VPTB: very preterm birth, EPTB: extremely preterm birth, SGA: small for gestational age, w: week(s).

**Table 2:** Definitions of birth outcomes and studied population (order by outcome).

	Type(s) or Subtype(s)	Outcome Classification	Population Study	Sample Size (Studies Population)	Database Study	Authors, Date
Birth weight			All singleton newborn Exclusion criteria Women <16 years of age, who not visited the public health center of Sabadell in the 12th week of pregnancy and not planning to deliver at the Hospital of Sabadell (followed an assisted reproduction program)	570 newborns	Cohort of women’s attendance at prenatal care in the public health center of Sabadell	Aguilera et al., 2009, [67]
			All live singleton newborns	785 newborns PTB: 51	INMA (INfancia y Medio Ambiente) cohort in Valencia, pregnant women attending the prenatal population-based screening program at the reference hospital	Ballester et al., 2010, [68]
			All live twins without congenital malformation excluded twins with missing data (birthweight, gestational age, zygosity, maternal age, parity)	4760 newborns	East Flanders Prospective Twin Survey (EFPTS) a population-based register of multiple births in the province of East Flanders (Belgium)	Bijnens et al., 2016, [82]
			Singleton births	13,775 newborns, 12,467 mothers	Aberdeen Maternity and Neonatal Databank (AMND) which has archived routinely acquired data from clinical activity at Aberdeen Maternity Hospital (AMH) since 1950	Clemens et al., 2017, [88]
			All newborn from January 2004 through December 2006 (from woman born in Italia and living in Lombardy)	3614 newborns	Clinica Mangiagalli, the largest maternity clinic in Milan	Giovannini et al., 2018, [89]
			Singleton term live births with at least 37 weeks with weight > 300 g registered between 1 January 1999 and 31 December 2002 Exclusion criteria: maternal address outside Oslo during the pregnancy, plural deliveries, term births with weight < 1000 g, or births with missing information on offspring’s gender or weight. Pregnancies with missing exposure on ambient air pollution from the dispersion model were also Excluded.	25,229 newborns LBW: 303 SGA: 2422	Medical Birth Registry of Norway (MBRN)	Madsen et al., 2010, [70]
			All singleton live births between February 2003 and January 2006 Exclusion criteria: all multiple fetuses, diabetes or planning to deliver outside the university hospital or to move out from the study region within 3 years of recruitment	1154 newborns	EDEN (Etudes des Déterminants pré et postnatals précoces du développement et de la santé de l’ENfant) mother–child cohort maternity records	Rahmalia et al., 2012, [72]
			All singleton live births between March 2007 and November 2010 Exclusion criteria: stillbirths, multiple pregnancies, infants whose maternal ethnic origin was not white British or Pakistani origin.	9067 newborns	Medical records of Bradford Royal Infirmary Born in Bradford (BiB)	Schembari et al., 2015, [80]
			Mothers enrolled before 26 gestational weeks at maternity wards of Nancy and Poitiers university hospitals, from 2002 to 2005 Exclusion criteria: women diabetes, multiple pregnancy, or intention to deliver outside the university hospital or to move out of the study area within 3 years.	1026 newborns (NO_2_ study area: 776 newborn)	EDEN mother–child cohort maternity records	Sellier et al., 2014, [76]
			All singleton live births between January 1998 and January 1999. Exclusion criteria: women diabetes), long-term use of medication, birth weight < 2500 g, gestational duration < 37 completed weeks, congenital malformation, symptomatic neonatal infection, antibiotic medication, and hospitalization or intensive medical care during neonatal period, twin births and women who changed home during pregnancy.	1016 newborns <3000: 142	Munich LISA (Influences of Lifestyle Related Factors on the Human Immune System and Development of Allergies in Children) birth cohort.	Slama et al., 2007, [66]
LBW	LBW < 2500	International Classification of Diseases 10th Revision (ICD-10): P07.0–P07.1),	All live singleton births from 1 January 2001 to 31 December 2009 (woman living in Madrid)	298,705 newborns LBW: 39,583	Madrid Regional Directorate-General of Economic Statistics and Technological Innovation	Arroyo et al., 2016, [81]
			Singleton term births (i.e., gestational age at delivery ≥37 weeks) occurring at the obstetrics department of the Hospital Clinic de Barcelona between January 2001 and June 2005 to mothers residing in the city of Barcelona.	6438 newborns Term LBW: 190	Cohort based on the data collected from Hospital Clinic de Barcelona	Dadvand et al., 2014, [75]
			All live singleton full term birth in the period 1 January 2001 to 31 December 2009 to whose mothers resided in the Madrid city area	298,705 newborns LBW: 3290 Term LBW: 1492	Perinatal health databases of public hospitals in Madrid	Diaz et al., 2016, [84]
			Singleton term live births with at least 37 weeks with weight >300 g registered between 1 January 1999 and 31 December 2002 Exclusion criteria: maternal address outside Oslo during the pregnancy, plural deliveries, term births with weight <1000 g, or births with missing information on offspring’s gender or weight. Pregnancies with missing exposure on ambient air pollution from the dispersion model were also Excluded.	25,229 newborns LBW: 303	Medical Birth Registry of Norway (MBRN)	Madsen et al., 2010, [70]
			All singleton births from 1 January 1998 through 31 December 1998	3988 newborns LBW: 140	Lithuanian National Birth Register	Maroziene and Grazuleviciene, 2002, [64]
			Singletons births between 11 February 1994, and 2 June 2011 and for whom information about home addresses during pregnancy, infant birthweight, gestational age, and sex was available (pooled data from 14 European mother–child cohort studies in which birthweight was not part of inclusion criteria)	74,178 newborns LBW: 1257	European Study of Cohorts for Air Pollution Effects (ESCAPE): data from 14 European mother–child cohort studies, MoBa (Norway); BAMSE (four centers; Sweden); DNBC (Denmark); KANC (Lithuania); BiB (England); ABCD, GENERATION R, and PIAMA (three centers; Netherlands); DUISBURG (Germany); EDEN (two centers; France), APREG (Hungary); GASPII (Italy); INMA (five centers; Spain); and RHEA (Greece)	Pedersen et al., 2013, [73]
	LBW < 3000		All non-premature singleton live births between January 1998 and January 1999. Exclusion criteria: women diabetes), long-term use of medication, birth weight < 2500 g, gestational duration < 37 completed weeks, congenital malformation, symptomatic neonatal infection, antibiotic medication, and hospitalization or intensive medical care during neonatal period, twin births and women who changed home during pregnancy.	1016 newborns LBW: 142	Munich LISA (Influences of Lifestyle Related Factors on the Human Immune System and Development of Allergies in Children) birth cohort.	Slama et al., 2007, [66]
	VLBW 1500–2500		All live singleton full term birth in the period 1 January 2001 to 31 December 2009 to whose mothers resided in the Madrid city area	298,705 newborns VLBW: 1176	Perinatal health databases of public hospitals in Madrid	Diaz et al., 2016, [84]
	ELBW <1500		All live singleton full term birth in the period 1 January 2001 to 31 December 2009 to whose mothers resided in the Madrid city area	298,705 newborns ELBW: 236	Perinatal health databases of public hospitals in Madrid	Diaz et al., 2016 [84]
PTB	PTB < 37	ICD-10: P07.2–P07.3	All live singleton births from 1 January 2001 to 31 December 2009	298,705 newborns PTB: 24,586 births	Madrid Regional Directorate-General of Economic Statistics and Technological Innovation	Arroyo et al., 2016, [81]
			All birth registered in the period of nine years from 2001 through 2009.	1468,622 newborns PTB: 127,722	National Statistics Institute (INE, 2018)	Arroyo et al., 2019, [92]
			Singleton live born infants without any major congenital malformation from 2002 to 2006	2509 newborns PTB: 83	PELAGIE cohort	Bertin et al., 2015, [78]
			Live births registered over the period 2008–2011	105,346 newborns PTB: 4871	First birth certificate information registered by Maternal and Child Care department of Paris	Deguen et al., 2018, [90]
			Singleton live birth recruited between November 2003 and February 2008	2409 newborns PTB: 115	main public hospital or reference health center in four study areas: Asturias, Gipuzkoa, Sabadell, and Valencia	Estarlich et al., 2016, [85]
			Live singleton births more than 24 weeks from 1988 to 2000 Exclusion criteria: babies weighing 200 g or less were either associated with a gestational age of less than 24 weeks or considered an error, congenital anomalies	482,765 newborns PTB: 29,716	St. Mary’s Maternity Information System (SMMIS)	Lee et al., 2007, [65]
			Live singleton births between February 2004 and June 2005. Women attending the prenatal population-based screening program at their referring hospital who met the inclusion criteria (In their first trimester, subjects had to reside in the study area, be at least 16 years old, have a singleton pregnancy, have their first prenatal visit in the main public hospital or health center of the area, not have followed any program of assisted reproduction, intend to deliver in the reference hospital, and have no communication problems)	785 newborns PTB: 47	INMA cohort in Valencia	Llop et al., 2010, [69]
			All singleton births from 1 January 1998 through 31 December 1998	3988 newborns PTB: 203	Lithuanian National Birth Register	Maroziene and Grazuleviciene, 2002, [64]
			Rome- All live singleton births (>22 w) between 1 April 2001 and the 31 October 2010. Barcelona- All live singleton births (>24 w) between 1 April 2007 and 31 October 2012. Exclusion criteria: multiple births, all cesarean sections where spontaneous onset of labor was not reported, labor inductions, births referred with congenital malformations, and stillbirths. Mothers younger than 11 years or older than 55 years.	78,633 newborns (Rome) 27,255 newborns (Barcelona) PTB in Rome: 4325 PTB in Barcelona: 1227	Cohort based on certificate of Delivery Care Registry for Rome and the Birth Registry of the Catalan Institute of Statistics in Barcelona	Schifano et al., 2016, [87]
			All newborn between 1 January 1984 and 31 March 1990. included all the children of the city of Espoo, Finland, born Random sample of children living in Espoo in 1991 from the roster of Statistics Finland.	2568 newborns PTB: 195	Espoo Cohort Study baseline data collection Finland’s Medical Birth Registry	Siddika et al., 2019, [93]
	PTB 33–37		Singleton live term births years 1994 to 2008 inclusive and birth weights ranging from 500 to 6000 g	21,843 newborns PTB: 1049	Scottish Longitudinal Study	Dibben et Clemens, 2015, [79]
	PTB 22–36		All singleton live births (>22 w) by natural delivery or cesarean sections with spontaneous onset of labor between 1 January 2001 and 31 December 2010 Exclusion criteria: multiple births, all cesarean sections where spontaneous onset of labor was not reported, labor inductions, births referred with congenital malformations, and stillbirths. Mothers younger than 11 years or older than 55 years.	132,691 newborns PTB 22–32 w: 847 PTB 33–36 w: 6412	Certificate of Delivery Care Registry Lazio regional hospital information system	Schifano et al., 2013, [74]
	PTB 30–37		All live singleton births between 1 January of 2001 and 31 December 2009	298,705 newborns PTB: 24,620 VPTB: 20,442 EPTB: 4178	Madrid Regional Directorate-General of Economic Statistics and Technological Innovation	Arroyo et al., 2015, [77]
	VPTB < 33		Singleton live term births years 1994 to 2008 inclusive and birth weights ranging from 500 to 6000 g	21,843 newborns VPTB: 193	Scottish Longitudinal Study	Dibben et Clemens, 2015, [79]
	VPTB < 30		All live singleton births between 1 January of 2001 and 31 December 2009	298,705 newborns PTB: 24,620 VPTB: 20,442 EPTB: 4178	Madrid Regional Directorate-General of Economic Statistics and Technological Innovation	Arroyo et al., 2015, [77]
	EPTB < 22		Rome-All live singleton births (>22 w) between 1 April 2001 and 31 October 2010. Barcelona- All live singleton births (>24 w) between 1f April 2007 and 31 October 2012. Exclusion criteria: multiple births, all cesarean sections where spontaneous onset of labor was not reported, labor inductions, births referred with congenital malformations, and stillbirths. Mothers younger than 11 years or older than 55 years.	78,633 newborns (Rome) 27,255 newborns (Barcelona) PTB in Rome: 4325 PTB in Barcelona: 1227	Cohort based on certificate of Delivery Care Registry for Rome and the Birth Registry of the Catalan Institute of Statistics in Barcelona	Schifano et al., 2016, [87]
	EPTB < 24		Rome-All live singleton births (>22 w) between 1 April 2001 and 31 October 2010. Barcelona- All live singleton births (>24 w) between 1 April 2007 and 31 October 2012. Exclusion criteria: multiple births, all cesarean sections where spontaneous onset of labor was not reported, labor inductions, births referred with congenital malformations, and stillbirths. Mothers younger than 11 years or older than 55 years.	78,633 newborns (Rome) 27,255 newborns (Barcelona) PTB in Rome: 4325 PTB in Barcelona: 1227	Cohort based on certificate of Delivery Care Registry for Rome and the Birth Registry of the Catalan Institute of Statistics in Barcelona	Schifano et al., 2016, [87]
SGA	Birth weight or length below the 10th percentile according to standard percentile charts for sex and gestational age in the population	ICD10 codes in medical records (O36.5, P05.0, P05.1)	All live singleton newborns	785 newborns PTB: 51	INMA cohort in Valencia	Ballester et al., 2010, [68]
			All live twins without congenital malformation	4760 newborns	East Flanders Prospective Twin Survey (EFPTS) a population-based register of multiple births in the province of East Flanders (Belgium)	Bijnens et al., 2016, [82]
			singleton term births (i.e., gestational age at delivery ≥37 w) occurring at the obstetrics department of the Hospital Clinic de Barcelona between January 2001 and June 2005 to mothers residing in the city of Barcelona.	6438 newborns Term LBW: 190 SGA: 803	Cohort based on the data collected from Hospital Clinic de Barcelona	Dadvand et al., 2014, [75]
			Singleton term live births with at least 37 weeks with weight > 300 g registered between 1 January 1999 and 31 December 2002 Exclusion criteria: maternal address outside Oslo during the pregnancy, plural deliveries, term births with weight < 1000 g, or births with missing information on offspring’s gender or weight. Pregnancies with missing exposure on ambient air pollution from the dispersion model were also Excluded.	25,229 newborns LBW: 303 SGA: 2422	Medical Birth Registry of Norway (MBRN)	Madsen et al., 2010, [70]
			Stillborn and live newborns, whose births occurred after 22 completed weeks of gestation and/or with birth weight > 500 g between 1 January 2005 and 31 December 2009	506 newborns SGA: 94	Besançon computerized medical records) and the Burgundy perinatal network records and paper medical records for Dijon	Mariet et al., 2018, [91]
Gestational age			All singleton newborn, exclusion criteria: women <16 years of age, who not visited the public health center of Sabadell in the 12th week of pregnancy and not planning to deliver at the Hospital of Sabadell (followed an assisted reproduction program)	570 newborns	Cohort of women’s attendance at prenatal care in the public health center of Sabadell	Aguilera et al., 2009, [67]

w: week(s), PTB: Preterm birth, VPTB: very preterm birth, EPTB: Extremely preterm birth, LBW: Low birth weight, VLBW: Very Low birth weight.

**Table 3:** Summary of approaches used to assess the residential exposure measures.

Approach	Database/Model Used	Pollutants	Indicators	Data Sources of Air Pollution	Level EXPOSURE Assigned to the Population	Authors, Date
Monitoring station-based approach						
**Average from all monitoring station**	monitoring stations of each province capital during the period 2001–2009	PM_10_, NO_2_	Weekly average	Ministry of Agriculture and Environment (MAGRAMA, n.d.)	Province capital level	Arroyo et al., 2019, [92]
	fixed monitoring stations at 53 different sites throughout the region.	PM_10_	Daily average	The Department of the Regional Environmental Protection Agency	Geographical area level	Giovannini et al., 2017, [89]
**Average from Monitoring stations existing**	27 urban background stations	PM_2.5_, NO_2_,	Daily mean	Madrid Municipal Air Quality Monitoring Grid	City level	Arroyo et al., 2016, [81]
	27 urban background stations	PM_2.5_, PM_10_, NO_2_,	Daily mean	Madrid Municipal Air Quality Monitoring Grid	City level	Arroyo et al., 2016, [77]
	27 urban background stations, gravimetric method	PM_2.5_, NO_2_,	Daily average	Madrid Municipal Air Quality Monitoring Grid	City level	Diaz et al., 2016, [84]
	One monitoring station located in Bloomsbury	PM_10_	Daily average	UK National Air Quality Archive	City level	Lee et al., 2007, [65]
	12 municipal monitoring sites, one in each residential district	NO_2_	Daily average,	Kaunas’ municipal ecological monitoring data	Residential district	Maroziene and Grazuleviciene, 2002, [64]
	three fixed stations in the urban area	PM_10,_ NO_2_	Daily mean	Lazio Environmental Protection Agency	City level	Schifano et al., 2013, [74]
	Rome, three fixed stations, one of background and two within the urban area Barcelona, data was obtained from a single urban background station	PM_10_, NO_2_	Daily mean	Rome, Lazio Environmental Protection Agency Barcelona, network of the Catalan Government	City level	Schifano et al., 2016, [87]
***Modeling based approach***						
**Modeling approaches**	LUR model, passive samplers and fix monitoring station	NO_2_	Daily mean		Individual level	Aguilera et al., 2009 [67]
	LUR model, passive samplers and fix monitoring station and kriging interpolation model	NO_2_	Daily average	Radiello^®^, Fondazione Salvatore Maugeri, Padua/Italy and monitoring network within 5 km or less of the study area	Individual level	Ballester et al., 2010, [68]
	LUR model, satellite and ground-based measurements and 12 monitoring station	NO_2_	Annual mean	The nationwide French NO_2_ concentrations European-wide NO_2_ concentrations (European APMoSPHERE project)	Individual level	Bertin et al., 2015, [78]
	spatial temporal interpolation method (Kriging) and monitoring stations	PM_10_, NO_2_	Daily mean	Corine land cover data set, Belgian telemetric air quality networks	Individual level	Bijnens et al., 2016, [82]
	Dispersion kernels model- Pollution Climate Mapping approach.	PM_2.5_, PM_10_, NO_2_	Annual mean	United Kingdom Department for the Environment, Food and Rural Affairs (DEFRA). National Atmospheric Emissions Inventory (NAEI)	Postcode level	Clemens et al., 2017, [88]
	LUR model and kriging interpolation method, passive samplers	NO_2_	Annual average	INMA: Radiello, Fundazione Salvatore Maugeri, Padua, Italy ENVIRONAGE: Belgian telemetric air quality network, point sources and line sources land cover data satellite images,	Individual level	Clemente et al., 2016, [83]
	LUR	PM_2.5_ PM_10_ NO_2_	Weekly exposure	European Study of Cohorts for Air Pollution Effects (ESCAPE)	Individual level	Dadvand et al., 2014, [75]
	Dispersion model- deterministic model. STREET dispersion model	NO_2_	Annual average	local air quality monitoring networks Airparif, The ESMERALDA inter-regional platform for air quality mapping and forecasting Emissions for traffic roads: COPERT III European database for the 2002–2006 period, and COPERT IV for the 2007–2012 period. meteorological data, Division of the NCAR Earth System Laboratory	Census block level	Deguen et al., 2018, [90]
	Dispersion kernel modelling- pollution climate mapping model approach	NO_2_, PM_10_	Annual average	United Kingdom Atomic Energy Authority (AEA) (now Ricardo-AEA), air quality by the UK government. National Atmospheric Emissions Inventory	Postcode level	Dibben et Clemens, 2015, [79]
	LUR and monitoring station, passive samplers	NO_2_	Daily mean	Radiellos, Fondazione Salvatore Maugeri, Padua, Italy	Individual level	Estarlich et al., 2016, [85]
	LUR, passive samplers and monitoring station	NO_2_	Daily mean	Radiellos, Fondazione Salvatore Maugeri, Padua, Italy	Individual level	Estarlich et al., 2011, [71]
	LUR and monitoring station	PM_2.5_ PM_10_ NO_2_	Annual mean		Individual level	Giorgis-Allemand et al., 2016, [86]
	kriging and LUR and monitoring station	NO_2_	Annual average (and daily variation)	Radiellos-type Valencia monitoring network	Individual level	Llop et al., 2010, [69]
	EPISODE, a dispersion model and monitoring station	NO_2_, PM_10_ PM_2.5_	Daily mean	Norwegian Institute for Air Research	Individual level (home and work address)	Madsen et al., 2010, [70]
	dispersion model	NO_2_	Monthly mean	traffic data using CIRCUL’AIR software, French Air Quality Monitoring Agencies COPERT IV European standard methodology	Individual level	Mariet et al., 2018, [91]
	LUR and monitoring station	PM_2.5_ PM_10_ NO_2_	Annual mean		Individual level	Pedersen et al., 2013, [73]
	Dispersion model implemented in ADMS-Urban software.	NO_2_, PM_10_	Hourly mean		Individual level	Rahmalia et al., 2012, [72]
	LUR models and monitoring station	PM_2.5_ PM_10_, NO_2_	Daily average	European Study of Cohorts for Air Pollution Effects	Individual level	Schembari et al., 2015, [80]
	Nearest AQMS model Temporally adjusted geostatistical model LUR model Dispersion model	NO_2_ PM_10_	Annual average	European Commission, Corine land cover 2006 (EEA 2005) Météo-France	Individual level	Sellier et al., 2014, [76]
	integrated modelling of atmospheric composition (SILAM)	PM _2.5_	Daily mean	Finnish Meteorological Institute	Individual level	Siddika et al., 2019, [93]
	LUR and monitoring station TRAPCA II model	PM _2.5_ NO_2_	Annual average	City of Munich	Individual level	Slama et al., 2007, [66]

NO_2_: nitrogen dioxide, PM: Particulate Matter; PM_2.5_: particulate matter with an aerodynamic diameter up to 2.5 μm; PM_10_: particulate matter with an aerodynamic diameter up to 10 μm.

**Table 4:** Definition and assessment of window of exposure.

	Windows of Exposure	Pollutants	Indicators	Authors
**Short-term exposure**				
**Daily exposure**	Lag 0	PM_10_	Daily average	Lee et al., 2007, [65]
		PM_10_ NO_2_	Daily mean	Schifano et al., 2013, [74]
	Lag 1	PM_10_ NO_2_	Daily mean	Schifano et al., 2013, [74]
	lags: 0 to lags 7 lagged days.	PM_2.5_, PM_10_, NO_2_	daily mean	Arroyo et al., 2015, [77]
		PM_10_ NO_2_	Daily mean	Schifano et al., 2013, [74]
	lags: 0 to lags 30 lagged days	PM_10_ NO_2_	Daily mean	Schifano et al., 2013, [74]
**Cumulative Exposure**	Over 1 days before birth (Lag 0–1)	PM_10_	Daily average	Lee et al., 2007, [65]
	Over 2 days before birth (Lag 0–2)	PM_10_	Daily average	Lee et al., 2007, [65]
		PM_10_, NO_2_	Daily mean	Schifano et al., 2016, [87]
	Over 3 days before the birth (Lag 0–3)	PM_10_	Daily average	Lee et al., 2007, [65]
	Over 4 days before the birth (Lag 0–4)	PM_10_	Daily average	Lee et al., 2007, [65]
	Over 5 days before birth (Lag 0–5)	PM_10_	Daily average	Lee et al., 2007, [65]
	Over 6 days before the birth (Lag 0–6)	PM_10_	Daily average	Lee et al., 2007, [65]
	Last week of pregnancy	PM_10_, NO_2_	Daily mean	Bijnens et al., 2016, [82]
**Long-term exposure**				
**Cumulative Exposure**	Weekly exposure	PM_2.5_, NO_2_	daily mean	Arroyo et al., 2016, [81]
		PM_10_, NO_2_	daily average	Arroyo et al., 2019, [92]
		PM_2.5_, PM_10_, NO_2_	Annual mean	Clemens et al., 2017, [88]
		PM_2.5_, NO_2_,	Daily average	Diaz et al., 2016, [84]
		PM_10_, NO_2_	Daily mean	Schifano et al., 2016, [87]
	7 week before	PM_2.5_, NO_2_,	Daily average	Diaz et al., 2016, [84]
	Last month of pregnancy	PM_10_, NO_2_	Daily mean	Bijnens et al., 2016, [82]
		PM_2.5_, NO_2_,	Daily average	Diaz et al., 2016, [84]
	2 months before delivery	NO_2_	Monthly mean	Mariet et al., 2018, [91]
	The first 2 trimester (t1-t2)	PM_2.5_ PM_10_ NO_2_	Annual mean	Giorgis-Allemand et al., 2016, [86]
	By trimester of pregnancy	NO_2_,	Daily mean Annual average	Aguilera et al., 2009, [67]
		NO_2_	daily average	Ballester et al., 2010, [68]
		PM_10_, NO_2_	Daily mean	Bijnens et al., 2016, [82]
		NO_2_	Annual average	Clemente et al.; 2016, [83]
		PM_2.5_ PM_10_ NO_2_	Weekly exposure	Dadvand et al., 2014, [75]
		NO_2_	Daily mean	Estarlich et al., 2016, [85]
		NO_2_	Daily mean	Estarlich et al., 2011, [71]
		PM_10_	Daily average	Giovannini et al., 2017, [89]
		NO_2_	Annual average (and daily variation)	Llop et al., 2010, [69]
		NO_2_, PM_10_ PM_2.5_	Hourly mean, Daily mean	Madsen et al., 2010, [70]
		NO_2_	Monthly mean	Mariet et al., 2018, [91]
		NO_2_	Daily average,	Maroziene and Grazuleviciene, 2002, [64]
		PM_2.5_ PM_10_ NO_2_	Annual mean (daily)	Pedersen et al., 2013, [73]
		NO_2_, PM_10_	Hourly mean	Rahmalia et al., 2012, [72]
		PM_2.5_ PM_10_, NO_2_	Daily average estimate	Schembari et al., 2015, [80]
		NO_2_ PM_10_	Annual average	Sellier et al., 2014, [76]
		PM_2.5_ NO_2_	Annual average	Slama et al., 2007, [66]
	During the 9 months of pregnancy	NO_2_	Daily mean, Annual average	Aguilera et al., 2009, [67]
		NO_2_	daily average	Ballester et al., 2010, [68]
		NO_2_	Annual average	Clemente et al.; 2016, [83]
		PM_2.5_ PM_10_ NO_2_	Weekly exposure	Dadvand et al., 2014, [75]
		NO_2_	Daily mean	Estarlich et al., 2016, [85]
		NO_2_	Daily mean	Estarlich et al., 2011, [71]
		PM_2.5_ PM_10_ NO_2_	Annual mean	Giorgis-Allemand et al., 2016, [86]
		NO_2_	Annual average (and daily variation)	Llop et al., 2010, [69]
		NO_2_, PM_10_ PM_2.5_	Hourly mean, daily mean	Madsen et al., 2010, [70]
		NO_2_	Monthly mean	Mariet et al., 2018, [91]
		NO_2_	Daily average	Maroziene and Grazuleviciene, 2002, [64]
		PM_2.5_ PM_10_ NO_2_	Annual mean (daily)	Pedersen et al., 2013, [73]
		NO_2_, PM_10_	Hourly mean	Rahmalia et al., 2012, [72]
		PM_2.5_ PM_10_ NO_2_	Daily average estimate	Schembari et al., 2015, [80]
		PM_10_, NO_2_	Daily mean	Schifano et al., 2016, [87]
		NO_2_ PM_10_	Annual average	Sellier et al., 2014, [76]
		PM_2.5_	Daily mean	Siddika et al., 2019, [93]
		PM_2.5_ NO_2_	Annual average	Slama et al., 2007, [66]
**No specific windows**	Annual exposure	NO_2_	Annual mean	Bertin et al., 2015, [78]
		NO_2_	Annual average	Deguen et al., 2018, [90]
		NO_2_, PM_10_	Annual average	Dibben et Clemens, 2015, [79]

PM: Particulate Matter; PM_10_: particulate matter with an aerodynamic diameter up to 10 μm; PM_2.5_: particulate matter with an aerodynamic diameter up to 2.5 μm; NO_2_: nitrogen.

**Table 5:** Definitions of measures of association for meta-analysis.

	Type(s) or Subtype(s)	Pollutants	Critical Windows	Model	Measure of Association	Mean Study Area	Authors, Date
**Birth weight**		**NO_2_**	**Trimester 1**	LUR	Beta = 3.3 (–33.2, 39.7)	First Trimester 32.66 μg/m^3^	Aguilera et al., 2009, [67]
					Beta = −12.782 (−34.5, 8.9)	37.9 μg/m^3^	Ballester et al., 2010, [68]
				Dispersion model implemented in ADMS-Urban software.	Beta = −3 (−42, 35)	24.9 μg/m^3^ in Nancy 16.1 μg/m^3^ in Poitiers	Rahmalia et al., 2012, [72]
				LUR and monitoring station	Odds ratio (OR) = 0.96 (0.73, 1.20)	35.8 µg/m^3^	Slama et al., 2007, [66]
				LUR. and kriging interpolation Method, land cover data satellite images	Beta = –44.1 (–77.4, –10.8)	INMA: 26.1 μg/m^3^ ENVIRONAGE: 20.7 μg/m^3^	Clemente et al.; 2016,), [83]
			**Trimester 2**	LUR	Beta = 3.7 (–31.1, 38.4)	2nd trimester 31.86 μg/m^3^	Aguilera et al., 2009, [67]
					Beta = −9.961 (−32.5, 12.6)	35.9 μg/m^3^	Ballester et al., 2010, [68]
				Dispersion model implemented in ADMS-Urban software.	Beta = 11 (−28, 50)	24.9 μg/m^3^ in Nancy 16.1 μg/m^3^ in Poitiers	Rahmalia et al., 2012, [72]
				LUR and monitoring station	OR = 1.18 (0.95, 1.44)	35.8 µg/m^3^	Slama et al., 2007, [66]
				LUR. and kriging interpolation Method, land cover data satellite images	Beta = –36.2 (–70.9, –1.6)	INMA: 25.6 μg/m^3^ ENVIRONAGE: 20.8 μg/m^3^	Clemente et al.; 2016,), [83]
			**Trimester 3**	LUR	Beta = 16.8 (–18.8, 52.4)	32.67 μg/m^3^	Aguilera et al., 2009, [67]
					Beta = −4.294 (−25.9, 17.3)	37 μg/m^3^	Ballester et al., 2010, [68]
				Dispersion model implemented in ADMS-Urban software.	Beta = −3 (−43, 37)	24.9 μg/m^3^ in Nancy 16.1 μg/m^3^ in Poitiers	Rahmalia et al., 2012, [72]
				LUR models and monitoring station	Beta = 4 (–13, 22)	21.4 µg/m^3^	Schembari et al., 2015, [80]
					OR = 1.13 (0.91, 1.35)	35.8 µg/m^3^	Slama et al., 2007, [66]
				LUR. and kriging interpolation Method, land cover data satellite images	Beta = –37.5 (–71.4, –3.6)	INMA: 25.7 μg/m^3^ ENVIRONAGE: 21.4 μg/m^3^	Clemente et al.; 2016, [83]
			**Whole pregnancy**	LUR	Beta = 8.8 (–23.8 to 41.5)	9 months 32.17 μg/m^3^	Aguilera et al., 2009, [67]
				LUR and kriging interpolation model and monitoring station	Beta = −9.729 (−33.218; 13.760)	36.9 μg/m^3^	Ballester et al., 2010, [68]
					Beta = –47.5 (–86.6, –8.5)	INMA: 25.5 μg/m^3^ ENVIRONAGE: 21.1 μg/m^3^	Clemente et al., 2016, [83]
				Dispersion model implemented in ADMS-Urban software.	Beta = 4 (−38 to 46)	24.9 μg/m^3^ in Nancy 16.1 μg/m^3^ in Poitiers	Rahmalia et al., 2012, [72]
				LUR models and monitoring station	Beta = −9 (–15, 34)	21.4 µg/m^3^	Schembari et al., 2015, [80]
					OR = 1.21 (0.86, 1.68)	35.8 µg/m^3^	Slama et al., 2007, [66]
					Beta = –1 (–6, 4)	26.2 μg/m^3^	Pedersen et al., 2013, [73]
		**NO_2_ >40µg/m^3^**	**Trimester 1**	LUR and kriging interpolation model and monitoring station	Beta = −40.349 (−96.267; 15.568)	36.9 μg/m^3^	Ballester et al., 2010, [68]
			**Trimester 2**	LUR and kriging interpolation model and monitoring station	Beta = −37.546 (−96.231; 21.140)	36.9 μg/m^3^	Ballester et al., 2010, [68]
			**Trimester 3**	LUR and kriging interpolation model and monitoring station	Beta = 26.656 (−28.239; 81.551)	36.9 μg/m^3^	Ballester et al., 2010, [68]
			**Whole pregnancy**	LUR and kriging interpolation model and monitoring station	Beta = −33.292 (−84.874; 18.290)	36.9 μg/m^3^	Ballester et al., 2010, [68]
		**PM_10_**	**Trimester 1**	Network of fixed monitoring stations at 53 different sites throughout the Lombardy region, Northern Italy and representatively distributed in eight geographical areas	Beta = −22.2 (−35.7, −8.7)	51.0 µg/m^3^	Giovannini et al., 2017, [89]
				Dispersion model implemented in ADMS-Urban software.	Beta = −8 (−104–88)	23.3 μg/m^3^ in Nancy 16.2 μg/m^3^ in Poitiers	Rahmalia et al., 2012, [72]
			**Trimester 2**	Network of fixed monitoring stations at 53 different sites throughout the Lombardy region, Northern Italy and representatively distributed in eight geographical areas	Beta = −10.1 (−24.2, 4.0)	51.0 µg/m^3^	Giovannini et al., 2017, [89]
				Dispersion model implemented in ADMS-Urban software.	Beta = −4 (−105, 97)	23.3 μg/m^3^ in Nancy 16.2 μg/m^3^ in Poitiers	Rahmalia et al., 2012, [72]
			**Trimester 3**	Network of fixed monitoring stations at 53 different sites throughout the Lombardy region, Northern Italy and representatively distributed in eight geographical areas	Beta = −5.1 (−18.4, 8.2)	51.0 µg/m^3^	Giovannini et al., 2017, [89]
				Dispersion model implemented in ADMS-Urban software.	Beta = −18 (−116 to 80)	23.3μg/m^3^ in Nancy 16.2 μg/m^3^ in Poitiers	Rahmalia et al., 2012, [72]
				LUR models and monitoring station	Beta = –13 (–42, 16)	21.4 µg/m^3^	Schembari et al., 2015, [80]
			**Whole pregnancy**	Dispersion model implemented in ADMS-Urban software.	Beta = −6 (−124 to 111)	23.3μg/m^3^ in Nancy 16.2 μg/m^3^ in Poitiers	Rahmalia et al., 2012, [72]
				LUR models and monitoring station	Beta = –9 (–41, 23)	21.4 µg/m^3^	Schembari et al., 2015, [80]
					Beta = –8 (–19, 3)	25.4 μg/m^3^	Pedersen et al., 2013, [73]
		**PM_2.5_**	**Trimester 3**	LUR models and monitoring station	Beta = –12 (–33, 8)	12.7 μg/m^3^	Schembari et al., 2015, [80]
			**Whole pregnancy**	LUR models and monitoring station	Beta = –7 (–17, 2)	16.5 μg/m^3^	Pedersen et al., 2013, [73]
					Beta = –11 (–33, 1)	12.7 μg/m^3^	Schembari et al., 2015, [80]
**PTB**		**NO_2_**	**Trimester 1**	LUR and monitoring station	OR = 1.02 (0.61–1.71)	28.8 μg/m^3^	Estarlich et al., 2016, [85]
					OR = 0.97 (0.92, 1.02)	Missing information	Giorgis-Allemand et al., 2016, [86]
				12 municipal monitoring sites, one in each residential district	OR = 1.67 (1.28, 2.18)	11.69 µg/m^3^	Maroziene and Grazuleviciene, 2002, [64]
			**Trimester 2**	LUR and monitoring station	OR = 1.06 (0.86–1.32)	28.8 μg/m^3^	Estarlich et al., 2016, [85]
					OR = 0.96 (0.92, 1.01)	Missing information	Giorgis-Allemand et al., 2016, [86]
				12 municipal monitoring sites, one in each residential district	OR = 1.13 (0.90, 1.40)	11.69 µg/m^3^	Maroziene and Grazuleviciene, 2002, [64]
			**Trimester 3**	LUR and monitoring station	OR = 1.02 (0.81–1.27)	28.8 μg/m^3^	Estarlich et al., 2016, [85]
				12 municipal monitoring sites, one in each residential district	OR = 1.19 (0.96, 1.47)	11.69 µg/m^3^	Maroziene and Grazuleviciene, 2002, [64]
			**Whole pregnancy**	LUR and monitoring station	OR = 1.11 (0.86–1.45)	28.8 μg/m^3^	Estarlich et al., 2016, [85]
					OR = 0.96 (0.91, 1.01)	Missing information	Giorgis-Allemand et al., 2016, [86]
				12 municipal monitoring sites, one in each residential district	OR = 1.25 (1.07, 1.46)	11.69 µg/m^3^	Maroziene and Grazuleviciene, 2002, [64]
				integrated modelling of atmospheric composition (SILAM)	OR = 0.83 (0.25, 2.74)	(ppb) 4.31	Siddika et al., 2019, [93]
			**Last Week**	LUR and monitoring station	OR = 0.98 (0.94, 1.01)	Missing information	Giorgis-Allemand et al., 2016, [86]
			**Last Month**	LUR and monitoring station	OR = 0.96 (0.92, 1.00)	Missing information	Giorgis-Allemand et al., 2016, [86]
		**PM_10_**	**Trimester 1**	LUR and monitoring station	OR = 0.98 (0.90, 1.07)	Missing information	Giorgis-Allemand et al., 2016, [86]
			**Trimester 2**	LUR and monitoring station	OR = 0.98 (0.90, 1.06)	Missing information	Giorgis-Allemand et al., 2016, [86]
			**Whole pregnancy**	LUR and monitoring station	OR = 0.97 (0.87, 1.07)	Missing information	Giorgis-Allemand et al., 2016, [86]
				Integrated modelling of atmospheric composition (SILAM)	OR = 0.98 (0.74, 1.31)	21.35 µg/m^3^	Siddika et al., 2019, [93]
			**Last week**	LUR and monitoring station	OR = 0.99 (0.95, 1.04)	Missing information	Giorgis-Allemand et al., 2016, [86]
			**Last month**	LUR and monitoring station	OR = 0.97 (0.91, 1.03)	Missing information	Giorgis-Allemand et al., 2016, [86]
			**Lag 0**	One monitoring station located in Bloomsbury	OR = 1.00 (1.00, –1.00)	27 µg/m^3^ (red on study’s figure)	Lee et al., 2007, [65]
		**PM_2.5_**	**Trimester 1**	LUR and monitoring station	OR = 0.98 (0.91, 1.05)	Missing information	Giorgis-Allemand et al., 2016, [86]
			**Trimester 2**	LUR and monitoring station	OR = 0.96 (0.90, 1.03)	Missing information	Giorgis-Allemand et al., 2016, [86]
			**Whole pregnancy**	LUR and monitoring station	OR = 0.96 (0.87, 1.04)	Missing information	Giorgis-Allemand et al., 2016, [86]
				Integrated modelling of atmospheric composition (SILAM)	OR = 1.00 (0.72, 1.38)	19.62 µg/m^3^	Siddika et al., 2019, [93]
			**Last week**	LUR and monitoring station	OR = 1.00 (0.96, 1.03)	Missing information	Giorgis-Allemand et al., 2016, [86]
			**Last month**	LUR and monitoring station	OR = 0.97 (0.91, 1.02)	Missing information	Giorgis-Allemand et al., 2016, [86]
			**Lag 20**	Network of 27 urban background stations	OR = 1.026 (1.018, 1.034)	17.1 µg/m^3^	Arroyo et al., 2016, [81]
			**Lag 1**	Network of 27 urban background stations	OR = 1.038 (1.002, 1.074)	17.1 µg/m^3^	Arroyo et al., 2015, [77]
**LBW**		**NO_2_**	**Trimester 1**	LUR	OR = 1.06 (0.94, 1.20)	Median pregnancy: 55.5 µg/m^3^	Dadvand et al., 2014, [75]
				12 municipal monitoring sites, one in each residential district	OR = 0.91 (0.53, 1.56)	11.69 µg/m^3^	Maroziene and Grazuleviciene, 2002, [64]
			**Trimester 2**	LUR	OR = 1.04 (0.91, 1.18)	Median pregnancy: 55.5 µg/m^3^	Dadvand et al., 2014, [75]
				12 municipal monitoring sites, one in each residential district	OR = 0.93 (0.61, 1.41)	11.69 µg/m^3^	Maroziene and Grazuleviciene, 2002, [64]
			**Trimester 3**	LUR	OR = 1.03 (0.90, 1.18)	Median pregnancy: 55.5 µg/m^3^	Dadvand et al., 2014, [75]
				12 municipal monitoring sites, one in each residential district	OR = 1.34 (0.94, 1.92)	11.69 µg/m^3^	Maroziene and Grazuleviciene, 2002, [64]
			**Whole pregnancy**	LUR	OR = 1.05 (0.94, 1.17)	Median pregnancy: 55.5 µg/m^3^	Dadvand et al., 2014, [75]
				12 municipal monitoring sites, one in each residential district	OR = 1.28 (0.97, 1.68)	11.69 µg/m^3^	Maroziene and Grazuleviciene, 2002, [64]
				LUR and monitoring station	OR = 1.09 (1.00, 1.19)	26.2 µg/m^3^	Pedersen et al., 2013, [73]
			**Lag 14**	Network of 27 urban background stations	OR = 1.011 (1.007, 1.014)	59.4 µg/m^3^	Arroyo et al., 2016, [81]
			**Lag 20**	Network of 27 urban background stations	OR = 1.014 (1.011, 1.017)	59.4 µg/m^3^	Arroyo et al., 2016, [81]
		**PM_10_**	**Trimester 1**	LUR	OR = 1.00 (0.82, 1.22)	Median pregnancy: 39.2 µg/m^3^	Dadvand et al., 2014, [75]
			**Trimester 2**	LUR	OR = 1.20 (0.96, 1.48)	Median pregnancy: 39.2 µg/m^3^	Dadvand et al., 2014, [75]
			**Trimester 3**	LUR	OR = 1.26 (1.06, 1.51)	Median pregnancy: 39.2 µg/m^3^	Dadvand et al., 2014, [75]
			**Whole pregnancy**	LUR	OR = 1.16 (0.98, 1.37)	Median pregnancy: 39.2 µg/m^3^	Dadvand et al., 2014, [75]
				LUR and monitoring station	OR = 1.16 (1.00, 1.35)	25.4 µg/m^3^	Pedersen et al., 2013, [73]
		**PM_2.5_**	**Trimester 1**	LUR	OR = 1.07 (0.88, 1.29)	Median pregnancy 16.9 µg/m^3^	Dadvand et al., 2014, [75]
			**Trimester 2**	LUR	OR = 1.19 (0.97, 1.45)	Median pregnancy 16.9 µg/m^3^	Dadvand et al., 2014, [75]
			**Trimester 3**	LUR	OR = 1.24 (1.03, 1.49)	Median pregnancy 16.9 µg/m^3^	Dadvand et al., 2014, [75]
			**Whole pregnancy**	LUR	OR = 1.17 (0.98, 1.39)	Median pregnancy 16.9 µg/m^3^	Dadvand et al., 2014, [75]
				LUR and monitoring station	OR = 1.18 (1.06, 1.33)	16.5 µg/m^3^	Pedersen et al., 2013, [73]
**SGA**		**NO_2_**	**Trimester 1**	LUR and kriging interpolation model and monitoring station	OR = 1.182 (0.894; 1.563)	37.9 μg/m^3^	Ballester et al., 2010, [68]
				dispersion model	OR = 0.78 (0.55, 1.12)	23.1 μg/m^3^	Mariet et al., 2018, [91]
			**Trimester 2**	LUR and kriging interpolation model and monitoring station	OR = 1.369 (1.013; 1.849)	35.9 μg/m^3^	Ballester et al., 2010, [68]
				dispersion model	OR = 0.83 (0.58, 1.19)	23.1 μg/m^3^	Mariet et al., 2018, [91]
			**Trimester 3**	LUR and kriging interpolation model and monitoring station	OR = 1.186 (0.906; 1.552)	37 μg/m^3^	Ballester et al., 2010, [68]
				dispersion model	OR = 0.88 (0.62, 1.25)	23.1 μg/m^3^	Mariet et al., 2018, [91]
			**Whole pregnancy**	LUR and kriging interpolation model and monitoring station	OR = 1.281 (0.942; 1.743)	36.9 μg/m^3^	Ballester et al., 2010, [68]
				Dispersion model	OR = 0.81 (0.56, 1.17)	23.1 μg/m^3^	Mariet et al., 2018, [91]
			**Las two month**	Dispersion model	OR = 0.88 (0.62, 1.25)	23.1 μg/m^3^	Mariet et al., 2018, [91]

LUR: land-use regression, LBW: low birth weight, PTB: preterm birth, w: week(s), NO_2_: nitrogen dioxide, PM: particulate matter; PM_10_: particulate matter with an aerodynamic diameter up to 10 μm; PM_2.5_: particulate matter with an aerodynamic diameter up to 2.5 μm, ADMS: Atmospheric Dispersion Modelling System.

**Figure A1:**
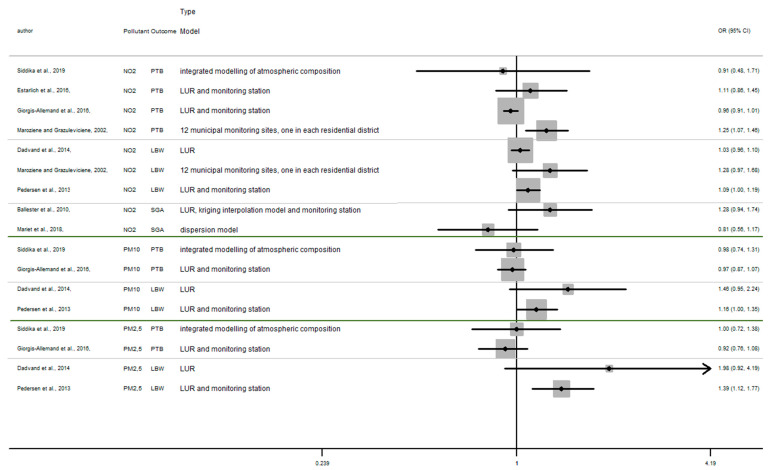
Risk of birth outcome for air pollutant exposure during overall pregnancy.

**Figure A2:**
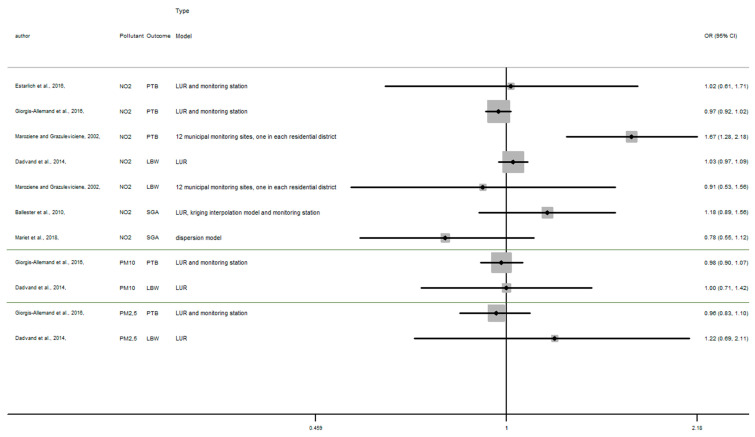
Risk of birth outcome for air pollutant exposure during first trimester.

**Figure A3:**
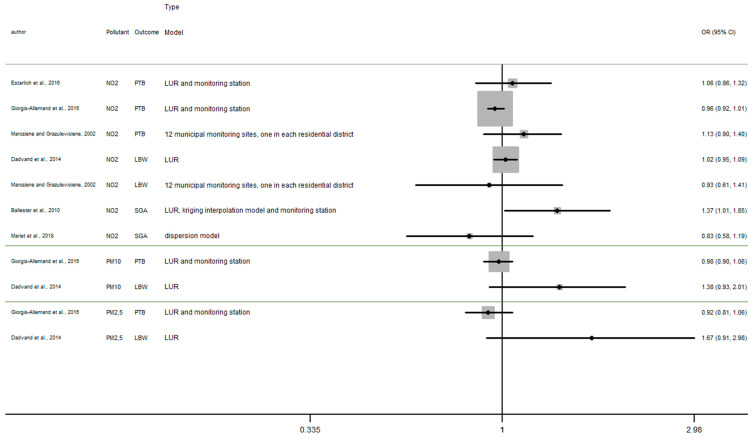
Risk of birth outcome for air pollutant exposure during second trimester.

**Figure A4:**
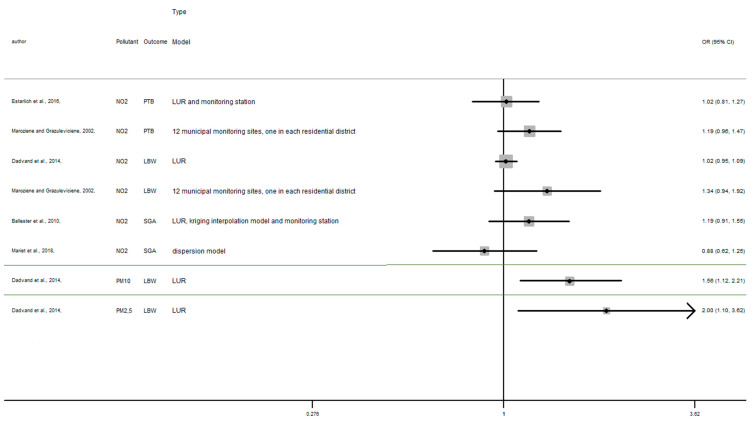
Risk of birth outcome for air pollutant exposure during third trimester.

## Appendix E

**Table A1:** Sensitivity analysis: birth weight and NO_2_ exposure during the third trimester of pregnancy.

Study Omitted	Beta	[95% Confidence Intervals]	
Aguilera et al., 2009 [67]	−4.29	−16.48	7.60
Ballester et al., 2010 [68]	−0.82	−13.74	12.10
Rahmalia et al., 2012 [72]	−1.63	−13.18	9.92
Schembari et al., 2015 [80]	−5.59	−19.93	8.76
Clemente et al., 2016 [83]	2.55	−9.18	14.30
Pooled estimate	−1.95	−14.50	10.54

**Table A2:** Sensitivity analysis: birth weight and NO_2_ exposure during the whole pregnancy.

Study Omitted	Beta	[95% Confidence Intervals]	
Aguilera et al., 2009 [67]	−1.60	−6.33	3.13
Ballester et al., 2010 [68]	−1.05	−5.83	3.73
Clemente et al.; 2016 [83]	−0.72	−5.44	3.99
Rahmalia et al., 2012 [72]	−1.47	−6.18	3.25
Schembari et al., 2015 [80]	−1.79	−6.57	2.98
Pedersen et al., 2013 [73]	−4.26	−17.69	9.16
Pooled estimate	−1.81	−8.01	4.37

## Appendix F

**Text. Quality effect model methods**

Individual quality assessment methodology was adapted from Croteau et al. in 2009 and doi and Thalib in 2008. The checklist was defined by researcher consensus. It assigned a maximum of 1.00 point for the different methodological criteria and a quality score (Qi = $(\;\frac{\sum_{1}^{10}score\;criteria}{10}$)) is calculated for each study included in the meta-analysis.

Ten criteria are defined as follows:

1- Sample size
  (1): completely satisfactory/justified by power analysis; (0.5): somewhat satisfactory; (0): not sufficient/not justified.
2- Design
  (1): cohort; (0.75): case-crossover; case-control; (0.5): ecological; time series
3- Country where the study was carried out
  (1): With good working and living conditions/high socio-economic standard; (0.5): Difficult conditions/lower socio-economic standard; (0.25): Very difficult conditions/very low socio-economic standard; (0): Not reported.
  (1: USA, UK, Sweden, Latin America; 0.5: China)
4- Timeframe
  (1): Reported; (0): Not reported.
5- Geocodage rate
  (1): ≥80%/considerable part of the population; (0.75): Not reported.
6- Definition of infant death
  (1): infant death excluding death due to accident and external causes or specific cause death; (0.75): death among singleton birth or among term birth; (0.5): overall death.
7- Assessment of infant death
  (1): valid database; (0.5): self-report; (0): not specified.
8- Assessment of the exposure
  (1): individual measure; (0.75): fine spatial level (zip code, municipality, ward level) (0.5): country level
9- Adjustments for covariates (cov)
  (1): At least 1 (cov) in each of the three covariates (baby’s characteristic, mother’s characteristics, or meteorological condition), (0.75): At least 1 (cov) baby’s characteristic and at least 1 (cov) mother’s characteristic (or meteorological condition); 0.5): At least 1 (cov) in meteorological condition; (0): no covariates
10- Effect size calculation for meta-analysis based on odds ratios
  (1): no transformations and no data imputation; (0.75): mild transformation and no data imputation; (0.5): several transformations and no data imputation; (0.25): considerable transformations and data imputation

**Table A3:** Qualitative analysis (part 1).

Auteurs	Aguilera et al., 2009 [67]	Ballester et al., 2010, [68]	Clemente et al., 2016). [83]	Pedersen et al., 2013 [73]	Rahmalia et al., 2012, [72]	Schembari et al., 2015 [80]
**Population size**	570 (1)	785 (1)	376 (1)	74,178 (1)	1154 (1)	9067 (1)
**Study design, period location,**	cohort (1)	cohort (1)	cohort (1)	Cohort (1)	Cohort (1)	Cohort (1)
**Country**	Sabadel, Spain (1)	Valencia, Spain (1)	Spain, Belgium (1)	European country (1)	Poitiers, Nancy, France (1)	England (1)
**Timeframe**	2004–2006 (1)	2004–2005 (1)	2004–2008, 2010–2013 (1)	1994–2011 (1)	2003–2006 (1)	2007–2010 (1)
**Monitoring station or model**	LUR model, passive samplers and fix monitoring station (1)	Land-use regression model, kriging interpolation model and monitoring station (1)	land use regression and kriging interpolation Method (1)	LUR and monitoring station (1)	Dispersion model implemented in ADMS-Urban software. (1)	LUR models and monitoring station (1)
**Assessment of birthweight**	recorded by specially trained midwives at delivery (1)	recorded by specially trained midwives at delivery (1)	recorded by specially trained midwives at delivery (1)	recorded by specially trained midwives at delivery and self-report (0.5)	recorded by specially trained midwives at delivery (1)	recorded by specially trained midwives at delivery (1)
**Adjustments for personal covariates**	- Maternal characteristic: tobacco smoking during pregnancy, Passive smoking during pregnancy, parity, education, race/ethnicity, age, gestational age, height, pre-pregnancy weight - birth characteristics: child’s sex, - others: Season of conception, Paternal height, Paternal weight. (1)	- Maternal characteristic: lifestyle variables twice during their pregnancy, maternal age, pre-pregnancy weight, height, gestational weight gain, parity, education, smoking during pregnancy, country of origin, season of last menstrual period - birth characteristics: sex. - neighborhood characteristics: Socio-demographic characteristics, - others: environmental exposure, paternal height (1)	- Maternal characteristic: age, ethnicity, parity, smoking status, education, pre-pregnancy maternal BMI - birth characteristics: gestational age, sex, - others: season of birth (1)	- Maternal characteristic: parity, active smoking, and education - birth characteristics: sex, - (0.75)	- Maternal characteristic: height, pre-pregnancy weight, parity, age at end of education, second trimester smoking, active smoking. - birth characteristics: gestational duration, infant sex, - others: season of last menstrual period, center of recruitment (1)	- Maternal characteristic: ethnicity (for adjusted and stratified), age, height, pregnancy weight at first gynecological visit, parity, active smoking during pregnancy, education, and housing tenure - birth characteristics: sex, gestational age, 2-hr post load plasma glucose test - others: season of conception, (1)
**exposure level**	Individual level (1)	Individual level (1)	Individual level (1)	Individual level (1)	Individual level (1)	Individual level (1)
**Geocodage rate**	Not reported (0.75)	Not reported (0.75)	Not reported (0.75)	Not reported (0.75)	Not reported (0.75)	Not reported (0.75)
**Quality index (Qi)**	0.972	0.972	0.972	0.806	0.972	0.972

**Table A4:** Qualitative analysis (part 2).

Auteurs	Estarlich et al., 2016, [85]	Giorgis-Allemand et al., 2016, [86]	Maroziene and Grazuleviciene, 2002, [64]	Siddika et al., 2019, [93]
**Population size**	2409 (1)	71,493 (1)	3988 (1)	2568 (1)
**Study design, period location,**	cohort (1)	cohort (1)	cohort (1)	cohort (1)
**Country**	Asturias, Gipuzkoa, Sabadell and Valencia, Spain (1)	11 European countries (1)	Kaunas, Lithuania (1)	Espoo, Finland (1)
**Timeframe**	2003–2008 (1)	1994–2001 (1)	1998 (1)	1984–1990 (1)
**Monitoring station or model**	LUR and monitoring station (1)	LUR and monitoring station (1)	12 municipal monitoring sites, one in each residential district (0.5)	integrated modelling of atmospheric composition (SILAM) (1)
**Assessment of birthweight**	Medical data and Self-report (0.5)	Medical data and Self-report (0.5)	valid database (1)	valid database (1)
**Adjustments for personal covariates**	- Maternal characteristic: socio-economic status, Active smoking during pregnancy, maternal age - birth characteristics: infant’s sex, - neighborhood characteristics: socio-demographic characteristics, environmental exposures, zone of residence - others: parental season of delivery, (1)	- Maternal characteristic: age, education, mother alone, parity, smoking, height and weight, pregnancy hypertension - birth characteristics: sex, cesarean delivery - neighborhood characteristics: country - others: meteorological factors, season of conception, Outdoor temperature, humidity, and atmospheric pressure, (1)	- Maternal characteristic: parity, age, marital status, education, maternal and paternal smoking, - birth characteristics: gestational age - others season of birth (1)	- Maternal characteristic: age, smoking during pregnancy, exposure to environmental tobacco smoke during pregnancy, single parenthood - birth characteristics: sex - neighborhood characteristics: exposure to other air pollutants, family’s socioeconomic status, (1)
**exposure level**	Individual level (1)	Individual level (1)	Residential district (0.75)	Individual level (1)
**Geocodage rate**	Not reported (0.75)	Not reported (0.75)	Not reported (0.75)	Not reported (0.75)
**Quality index (Qi)**	0.917	0.917	0.889	0.972

## Appendix G

**Table A5:** Meta-analysis comparison.

First Author	Number of Study Included	Main Location	Main Design	Main Exposure Assessment	Pollutant	Outcomes		
						PTB OR (95%CI)	BW Beta (95%CI)	LBW OR (95%CI)
Li et al., 2017 [56]	23	USA	cohort design	ground-based monitoring data	PM_10_	NA	NA	NA
					PM_2.5_	1T 1.03 (1.00, 1.06)	NA	1T 1.00 (0.91, 1.11)
						2T 1.01 (0.93, 1.10)		2T 1.00 (0.96, 1.03)
						3T 1.02 (0.99, 1.04)		3T 1.03 (0.98, 1.09)
						EP 1.02 (0.93, 1.12)		EP 1.05 (0.98, 1.12)
						EP (IQR) 1.03 (1.01, 1.05)		EP (IQR) 1.03 (1.02, 1.03)
					NO_2_	NA	NA	NA
Stieb et al., 2012 [32]	61	North America	cohort design	central site monitoring data	PM_10_	1T 0.97 (0.87, 1.07)	1T −3.92 (−8.97, 1.13)	1T 1.03 (0.95, 1.11)
						2T 0.95 (0.91, 0.99)	2T −3.40 (−7.22, 0.43)	2T 1.02 (0.96, 1.09)
						3T 1.06 (1.03, 1.11)	3T −4.20 (−14.27, 5.86)	3T 1.01 (0.97, 1.06)
						EP 1.35 (0.97, 1.90)	EP −16.77 (−20.23, −13.31)	EP 1.10 (1.05, 1.15)
					PM_2.5_	1T 0.85 (0.60, 1.20) 3T 1.05 (0.98, 1.13) EP 1.16 (1.07, 1.26)	1T −0.30 (−9.85, 9.25) 2T −14.66 (−34.01, 4.70) 3T −18.05 (–37.43, 1.34) EP−23,44 (−45.50, −1.38)	EP 1.05 (0.99, 1.12)
					NO_2_	1T 0.87 (0.64, 1.17) 3T 1.06 (0.96, 1.18) EP 1.16 (0.83, 1.63)	1T −4.18 (−19, 10.82) 2T 0.85 (−1.27, 2.97) 3T −7.89 (−29.04, 13.25) EP −28.13 (−44.81, −11.45)	1T 1.03 (0.99, 1.14) 2T 1.04 (1.01, 1.08) 3T 0.98 (0.87, 1.10) EP 1.05 (1.00, 1.09)
Klepac et al., 2018 [26]	48	North America	cohort design	routine monitoring data	PM_10_	1T 1.04(1.01, 1.08)	NA	NA
						2T 1.04 (0.98, 1.09)		
						3T 1.00 (0.99, 1.00)		
						1M 1.05 (0.90, 1.24)		
						LM 1.01 (0.99, 1.03)		
						EP 1.09 (1.03, 1.16)		
					PM_2.5_	1T 1.03 (0.95, 1.11)	NA	NA
						2T 1.10 (0.96, 1.27)		
						3T 1.05 (1.02, 1.09)		
						1M 1.04 (0.91, 1.19)		
						LM 1.04 (0.98, 1.10)		
						EP 1.24 (1.08, 1.41)		
					NO_2_	1T 0.99 (1.95, 1.03)	NA	NA
						2T 1.02 (0.97, 1.08)		
						3T 1.02 (0.96, 1.08)		
						1M 0.91 (0.80, 1.04)		
						LM 1.03 (1.00, 1.05)		
						EP 1.05 (0.99, 1.11)		

PM_10_ and PM_2.5_: per 10 mg/m^3^ increment and 20 mg/m^3^ increment (depending on study) NO_2_ per 10 ppb increment, OR: odds ratio, PTB: preterm birth, BW: birthweight, LBW: low birth weight, PM: particulate matter; PM_10_: particulate matter with an aerodynamic diameter up to 10 μm; PM_2.5_: particulate matter with an aerodynamic diameter up to 2.5 μm; NO_2_: nitrogen, 1T: first trimester, 2T: second trimester, 3T: third trimester, 1M: first month, LM: last month, EP: entire pregnancy.

## Appendix H

**Table A6:** Interquartile range (IQR) table for NO_2_ exposure (µg/m^3^).

Studies	First Trimester IQR	Second Trimester IQR	Third Trimester IQR	Whole Pregnancy IQR
Aguilera et al., 2009 [67]	12.27	12	12.47	9.51
Ballester et al., 2010 [68]	10	10	10	10
Rahmalia et al., 2012 [72]	10	10	10	10
Schembari et al., 2015 [80]	NA	NA	10	10
Clemente et al., 2016 [83]	10	10	10	10
Estarlich et al., 2016, [85]	10	10	10	10
Maroziene and Grazuleviciene, 2002 [64]	10	10	10	10
Giorgis-Allemand et al., 2016 [86]	10	10	NA	10
Siddika et al., 2019 [93]	NA	NA	NA	18.8
Dadvand et al., 2014 [75]	20.5	19.9	18.7	16.8
Pedersen et al., 2013 [73]	NA	NA	NA	10
Mariet et al., 2018 [91]	10	10	10	10

**Table A7:** IQR table for PM_10_ exposure (µg/m^3^).

Studies	First Trimester IQR	Second Trimester IQR	Third Trimester IQR	Whole Pregnancy IQR
Rahmalia et al., 2012 [72]	10	10	10	10
Schembari et al., 2015 [80]	NA	NA	10	10
Giorgis-Allemand et al., 2016 [86]	10	10	NA	10
Siddika et al., 2019 [93]	NA	NA	NA	10
Dadvand et al., 2014 [75]	5.7	5.6	5.2	3.9
Pedersen et al., 2013 [73]	NA	NA	NA	10
Giovannini et al., 2017, [89]	10	10	10	NA

**Table A8:** IQR table for PM_2.5_ exposure (µg/m^3^).

Studies	First Trimester IQR	Second Trimester IQR	Third Trimester IQR	Whole Pregnancy IQR
Schembari et al., 2015 [80]	NA	NA	5	5
Giorgis-Allemand et al., 2016 [86]	5	5	NA	5
Siddika et al., 2019 [93]	NA	NA	NA	10
Dadvand et al., 2014 [75]	3.4	3.4	3.1	2.3
Pedersen et al., 2013 [73]	NA	NA	NA	5
